# Structural basis for the assembly of the Ragulator-Rag GTPase complex

**DOI:** 10.1038/s41467-017-01762-3

**Published:** 2017-11-20

**Authors:** Ryo Yonehara, Shigeyuki Nada, Tomokazu Nakai, Masahiro Nakai, Ayaka Kitamura, Akira Ogawa, Hirokazu Nakatsumi, Keiichi I. Nakayama, Songling Li, Daron M. Standley, Eiki Yamashita, Atsushi Nakagawa, Masato Okada

**Affiliations:** 1 Laboratory of Supramolecular Crystallography, Institute for Protein Research, Osaka University, 3-2 Yamadaoka, Suita, Osaka, 565-0871 Japan; 2 Department of Oncogene Research, Research Institute for Microbial Diseases, Osaka University, 3-1 Yamadaoka, Suita, Osaka, 565-0871 Japan; 3 Medical Institute of Bioregulation, Kyushu University, 3-1-1 Maidashi, Higashi-ku, Fukuoka, 812-0054 Japan; 4 Department of Genome Informatics, Research Institute for Microbial Diseases, Osaka University, 3-1 Yamadaoka, Suita, Osaka, 565-0871 Japan

## Abstract

The mechanistic target of rapamycin complex 1 (mTORC1) plays a central role in regulating cell growth and metabolism by responding to cellular nutrient conditions. The activity of mTORC1 is controlled by Rag GTPases, which are anchored to lysosomes via Ragulator, a pentameric protein complex consisting of membrane-anchored p18/LAMTOR1 and two roadblock heterodimers. Here we report the crystal structure of Ragulator in complex with the roadblock domains of RagA-C, which helps to elucidate the molecular basis for the regulation of Rag GTPases. In the structure, p18 wraps around the three pairs of roadblock heterodimers to tandemly assemble them onto lysosomes. Cellular and in vitro analyses further demonstrate that p18 is required for Ragulator-Rag GTPase assembly and amino acid-dependent activation of mTORC1. These results establish p18 as a critical organizing scaffold for the Ragulator-Rag GTPase complex, which may provide a platform for nutrient sensing on lysosomes.

## Abstract (web summary)

mTORC1 activity is controlled through Rag GTPases, which are anchored to the lysosome through the Ragulator. Here, the authors give molecular insights into Ragulator-Rag GTPase assembly and present the crystal structures of the Ragulator alone and in complex with the RagA-C roadblock domains.

## Introduction

The mechanistic target of rapamycin complex 1 (mTORC1) serves as a master regulator of cell growth and metabolism by controlling biomaterial synthesis, lysosome biogenesis, and autophagy^1–3^ (Supplementary Fig. 1). mTORC1 is a multiprotein complex consisting of mTOR, RAPTOR, DEPTOR, PRAS40, and mLST8^4^. mTOR is a highly conserved serine/threonine kinase that belongs to the family of phosphoinositide 3-kinase (PI3K)-related kinases and phosphorylates downstream substrates, such as S6 kinase, 4E-BP1, TFEB, and ULK1, to induce differential cellular responses depending upon nutrient conditions^4^. Dysregulation of critical components in the mTORC1 pathway is implicated in various human diseases such as cancer and diabetes-related complications. The mTORC1 pathway is therefore considered a promising therapeutic target for such diseases^5,6^.

The kinase activity of mTORC1 is regulated through multiple signals including nutrient amino acids, extracellular growth factors (e.g., insulin and insulin-like growth factors), and the intracellular energy status^7–11^. The sensing of nutrient amino acids by specific factors, such as SESTRIN2 and CASTOR1, leads to inactivation of GATOR1, a GTPase-activating protein (GAP) for Rag GTPase, resulting in the activation of Rag GTPase. Signals from other types of nutrient-sensing factors, such as LRS, FLCN-FNIP2, SLC38A9, and V-ATPase, also converge on Rag GTPase^11^. Activated Rag GTPase binds to mTORC1 and promotes its translocation to the surface of lysosomes^12,13^, whereupon mTORC1 is directly activated through interaction with another small GTPase, RHEB. The activity of RHEB is regulated by signals from growth factor receptors and by the cellular energy status. Activation of the PI3K-AKT pathway downstream of growth factor receptors induces inactivation of TSC1-2, a GAP for RHEB, leading to the activation of RHEB. By contrast, a decrease in cellular energy levels (i.e., a high AMP/ATP ratio) is sensed by AMP kinase^14^, which phosphorylates and activates TSC1-2 to suppress RHEB.

Rag GTPase is tethered to the surface of lysosomes via Ragulator^15,16^, a heteropentameric protein complex consisting of a membrane-anchored scaffold protein p18/LAMTOR1^17–20^ and two heterodimers composed of roadblock domains^21,22^: p14-MP1/LAMTOR2-3^23,24^ and p10-HBXIP/LAMTOR4-5^25^. Rag GTPase is a heterodimeric complex formed by RagA/B and RagC/D, both of which consist of nucleotide-binding domains and roadblock domains^21,26^. Interaction between Ragulator and Rag GTPase is required for mTORC1 activation on lysosomes, and Ragulator has been proposed to act as a guanine nucleotide exchange factor (GEF) for Rag GTPase^15,25,26^. However, the molecular basis for the function and nutrient-dependent regulation of Ragulator and Rag GTPase remains elusive owing to a lack of structural information.

Herein, we present crystal structures of Ragulator and Ragulator in complex with the roadblock domains of RagA-C [RagA(RD)-C(RD)]. These structures reveal that p18 functions as a flexible scaffold by wrapping around the three pairs of roadblock heterodimers [i.e., p14-MP1, p10-HBXIP, and RagA(RD)-C(RD)] to tandemly assemble them via head-to-tail interactions. Cellular and in vitro reconstitution assays demonstrated that p18 is required for the functional assembly of the Ragulator-Rag GTPase complex and amino acid-dependent activation of mTORC1. These results suggest that the module built from the three roadblock heterodimers may serve as a platform for additional factors involved in the nutrient-dependent regulation of mTORC1 on lysosomes.

## Results

### The structure of Ragulator

We first analyzed the crystal structure of Ragulator, a heteropentameric protein complex. To enable its crystallization, we identified the minimum region of p18 essential for Ragulator formation. Immunofluorescence analysis showed that the C-terminal 120 amino acid residues of p18 (Ser42–Pro161) were required for the assembly of a functional Ragulator on lysosomes (Supplementary Fig. 2a, b). Thus, this p18 fragment was N-terminally His-tagged and coexpressed with other components of Ragulator in *Escherichia coli* (Supplementary Fig. 2c). The heteropentameric complex was purified by sequential column chromatography, followed by crystallization (Supplementary Fig. 2d). X-ray diffraction data were collected to 2.40 Å resolution, and the structure of Ragulator was determined by molecular replacement (Table 1 and Supplementary Fig. 3).

**Table 1:** Data collection and refinement statistics

	Ragulator	Ragulator-RagA(RD)-C(RD)
PDB ID	5X6U	5X6V
*Data collection*		
Space group	*P*4_1_22	*P*2_1_2_1_2_1_
Cell dimensions		
*a*, *b*, *c* (Å)	133.04, 133.04, 75.15	74.10, 93.48, 125.45
*α*, *β*, *γ* (°)	90.0, 90.0, 90.0	90.0, 90.0, 90.0
Resolution (Å)	133.04–2.40 (2.44–2.40)*	125.45–2.02 (2.06–2.02)
*R*_merge_	0.060 (1.116)	0.069 (0.856)
*I/σ* (*I*)	23.9 (2.2)	13.6 (2.1)
*CC*_1/2_	1.000 (0.771)	0.998 (0.818)
Completeness (%)	100.0 (99.5)	99.4 (99.3)
Redundancy	10.9 (11.3)	5.5 (5.6)
*Refinement*		
Resolution (Å)	133.04–2.40 (2.44–2.40)	19.67–2.02 (2.06–2.02)
No. of reflections	26,910	57,304
*R*_work_/*R*_free_	18.60/23.16	19.25/24.34
No. of atoms		
Protein	3,799	6,064
Ligand/ion	0	5
Water	130	348
*B* factors (Å^2^)		
Protein	76.1	59.8
Ligand/ion	N/A	41.2
Water	81.1	63.2
R.m.s. deviations		
Bond lengths (Å)	0.010	0.010
Bond angles (°)	1.18	1.11
Ramachandran plot		
Favored (%)	97.1	98.0
Allowed (%)	2.5	2.0
Outliers (%)	0.4	0

*Values in parentheses are for the highest-resolution shell

**Fig. 1:**
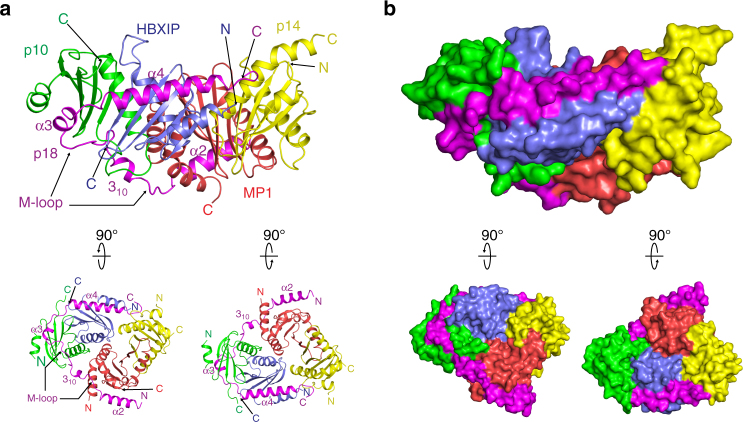
The overall structure of Ragulator. **a** Ribbon model representation of the crystal structure of Ragulator. p18, magenta; p14, yellow; MP1, red; p10, green; HBXIP, blue; N, amino terminus; C, carboxyl terminus. The locations of helices α2, 3_10_, α3 and α4, and the M-loop of p18 are shown (upper panel). The structure is viewed from two different angles rotated at 90° along the horizontal axis (lower panel). **b** Surface representation of Ragulator (upper panel). The structure is viewed from two different angles rotated at 90° along the horizontal axis (lower panel)

The overall structure of Ragulator revealed that the central region of p18 (Gln78–Ala150) wraps around two roadblock heterodimers: p14-MP1 and p10-HBXIP (Fig. 1a, b). Electron density for the N- and C-terminal regions (Ser42–Glu77 and Lys151–Pro161, respectively) was not visible, suggesting that these segments are flexible and disordered. These features of p18 indicate that it is an intrinsically disordered protein that adopts the observed structure by specifically interacting with the two heterodimers.

In the presence of roadblock domains, p18 adopts various secondary structural elements: it forms two long α-helices at the N- and C-terminal ends (helices α2 and α4, respectively), a short α-helix (α3), and a 3_10_ helix in the middle loop (M-loop) between the α2 and α3 helices (Fig. 1a). The α2 helix (His79–Val94) mainly interacts with the hydrophobic groove of MP1, and the M-loop is elongated and also interacts with the hydrophobic groove formed by p10 and MP1 (Supplementary Fig. 4a). In particular, the side chains of Leu108 and Leu111 in the proline-rich region of the M-loop (Pro106, Pro107, and Pro109) are buried in the hydrophobic pocket of p10 (Supplementary Fig. 4a). The α3 helix (Pro115–Leu119) of p18 extensively interacts with the β-sheets of p10, and is supported by multiple hydrogen bonds and hydrophobic interactions (Fig. 1a, b and Supplementary Figs. 4a and 5a). Ser121 and Glu122 of p18 form hydrogen bonds with Arg65 and Arg89 of p10, creating an Arg bridge that forms further hydrogen bonds with Val118 and Leu119 of p18 (Supplementary Fig. 5a). The α4 helix (Phe126–Leu143) of p18 tightly interacts with HBXIP to assemble a stable trimer (p18-p10-HBXIP) that provides a platform for p14-MP1 (Fig. 1a). The binding interface between p18 and HBXIP is composed of hydrophobic interactions and hydrogen bonds between Asp128, Gln131, Ser133, and Ile135 of p18, and Lys88, Asn15, Gln77, and His8 of HBXIP, respectively (Supplementary Figs. 4a and 5b). The C-terminal region of p18 weakly interacts with helix α1 of p14 (Fig. 1a, b). Through these interactions, p18 appears to assemble the two roadblock heterodimers into a stable tetramer.

### Assembly of p14-MP1 and p10-HBXIP heterodimers

**Fig. 2:**
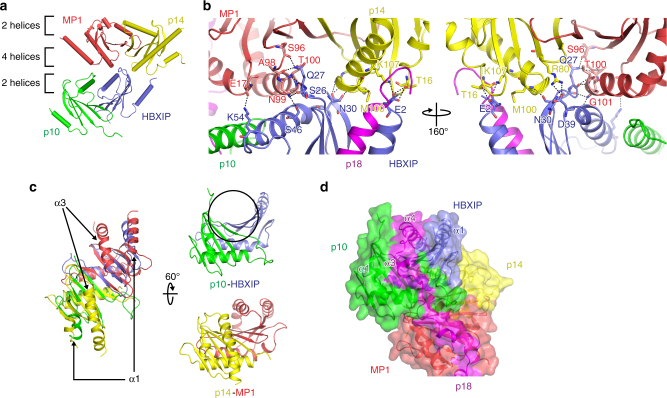
Assembly of p14-MP1 and p10-HBXIP heterodimers. **a** The orientations of roadblock heterodimers upon formation of the heterotetramer in the structure of Ragulator. The β1–β2 loop near the two helices docks into the groove formed by the α3 helix on the side of four helices. **b** Ribbon model representation of interactions between p14-MP1 and p10-HBXIP roadblock dimers (left panel). The structure is viewed from different angles rotated at 160° along the vertical axis (right panel). Hydrogen bonds are shown as black dashed lines. **c** Ribbon model representation of the p10-HBXIP dimer superimposed on the p14-MP1 dimer (left panel). The structures of each dimer are viewed from different angles rotated at 60° along the horizontal axis (right panel). The groove formed in the p10-HBXIP dimer is shown as circled. **d** Ribbon model representation of p18 superimposed on p14, MP1, p10, and HBXIP. Molecular surface models are also superimposed

The core region of Ragulator consists of two roadblock heterodimers, each with pseudo 2-fold symmetry, which are assembled tandemly in a head-to-tail orientation (Fig. 2a). HBXIP interacts with both p14 and MP1 (buried surface area [BSA] ~440 Å^2^ and ~360 Å^2^, respectively), while p10 contacts only with MP1 (BSA ~240 Å^2^; Fig. 2a and Supplementary Table 1). These BSAs are smaller than those between p14 and MP1, or between p10 and HBXIP (BSA ~1250 Å^2^ and ~830 Å^2^, respectively), suggesting that the interactions between the two heterodimers are weaker than those within each heterodimer, and indicating that wrapping by p18 is necessary to stabilize the interaction between the two heterodimers. The narrow binding interface between HBXIP and p14-MP1 is mediated by hydrogen bonds and by three salt bridges formed by Glu2, Asp39, and Lys54 of HBXIP with Arg80 and Lys107 of p14 and Glu17 of MP1 (Fig. 2b). These interactions may also contribute to the assembly of Ragulator.

The p10 subunit forms a heterodimer with HBXIP in essentially the same way as p14-MP1^27,28^ (Fig. 2c, left). In the p10-HBXIP heterodimer, the α2 helices of each component interact in an antiparallel fashion, and the β3 strands form an antiparallel β-sheet. However, in contrast to the p14-MP1 heterodimer, both components lack helix α3 (Fig. 2c, right), thereby leaving a hydrophobic groove along the outer face of the β-sheet, which facilitates interaction with the α3 and α4 helices of p18 (Fig. 2d).

### The structure of Ragulator-RagA-C roadblock domains

To elucidate the structural basis for the interaction between Ragulator and RagA-C, we next investigated which regions of RagA-C interact with Ragulator. For this, we coexpressed Ragulator with full-length RagA-C, the GTP-binding domains of RagA-C or the C-terminal roadblock dimers of RagA-C [RagA(RD)-C(RD)]. Pull-down assays with HisTrap beads revealed that RagA(RD)-C(RD) was sufficient to bind to Ragulator in vitro. Therefore, we used the full Ragulator along with RagA(RD)-C(RD) to determine the core structure of the Ragulator–RagA-C complex. All components of Ragulator and RagA(RD)-C(RD) were coexpressed in *E. coli*, and the purified heptameric complex was crystallized (Supplementary Fig. 2c, e). X-ray diffraction data were collected to 2.02-Å resolution, and the structure was determined by molecular replacement using structures of Ragulator and Gtr1p–Gtr2p^29^, a yeast ortholog of RagA-C (PDB: 4ARZ), as templates (Table 1 and Supplementary Fig. 6).

**Fig. 3:**
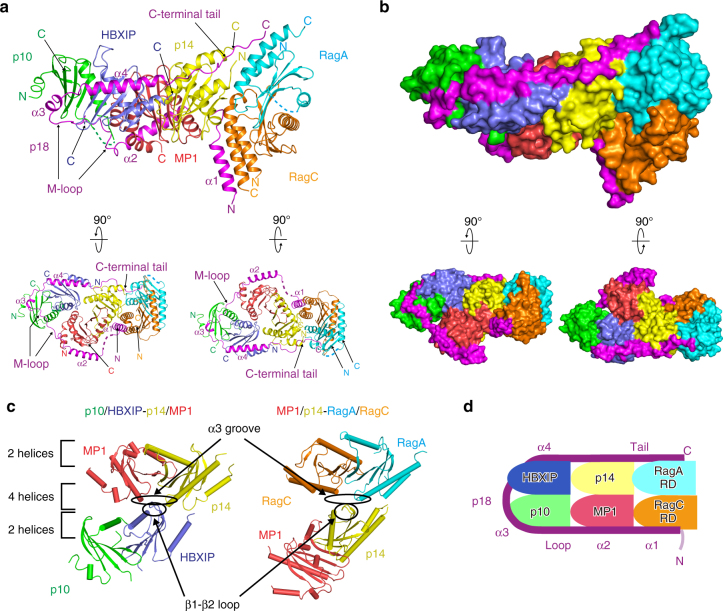
The overall structure of Ragulator in complex with RagA(RD)-C(RD). **a** Ribbon model representation of the crystal structure of the Ragulator-RagA(RD)-C(RD) complex. p18, magenta; p14, yellow; MP1, red; p10, green; HBXIP, blue; RagA(RD), cyan; RagC(RD), orange; N, amino terminus; C, carboxyl terminus. The locations of helices α1, α2, α3, and α4 helices, and the M-loop and C-terminal tail of p18, are shown (upper panel). The structure is viewed from two different angles rotated at 90° along the horizontal axis (lower panel). **b** Surface representation of Ragulator-RagA(RD)-C(RD) (upper panel). The structure is viewed from two different angles rotated at 90° along the horizontal axis (lower panel). **c** Orientations of roadblock heterodimers upon formation of the heterotetramer in the structure of Ragulator (left panel) and Ragulator-RagA(RD)-C(RD) (right panel). **d** Schematic model of the structure of the Ragulator-RagA(RD)-C(RD) complex

The overall structure of the Ragulator-RagA(RD)-C(RD) complex revealed that the N-terminal region of p18, which was disordered in the Ragulator structure, becomes ordered and clearly visible in the heptameric complex and forms a helix (α1: Glu50–Asn60), while the loop located between α1 and α2 remains disordered (Fig. 3a, b). The α1 helix interacts with the antiparallel pair of N- and C-terminal α helices of RagC(RD) through hydrophobic contacts (Supplementary Fig. 4b). This interaction is further stabilized by multiple hydrogen bonds between p18 (Ser63, Asn64, Ile65, Ile66, and Val68) and RagC (Asn252, Arg347, Tyr353, and Asn354) or MP1 (Lys62) (Supplementary Fig. 7a).

The C-terminal tail of p18 (Lys151–Pro161) is also visible, and is stabilized by hydrophobic contacts with p14 and RagA(RD) (Fig. 3a and c, Supplementary Fig. 4b). Furthermore, Glu152, Glu153, Leu154, and Val156 of p18 interact with Met1 and Arg3 of p14 through antiparallel β-strand-like interactions and the formation of multiple hydrogen bonds (Supplementary Fig. 7b). These interactions are observed only in the structure of Ragulator-RagA(RD)-C(RC), indicating a crucial role of the C-terminal tail of p18 for the assembly of Ragulator-RagA(RD)-C(RD).

The roadblock domains of RagA-C form a heterodimer with a pseudo 2-fold symmetry in a similar manner to that observed in the p14-MP1 and p10-HBXIP dimers. The binding mode between RagA(RD)-C(RD) and p14-MP1 is similar to that between p14-MP1 and p10-HBXIP: the β1–β2 loop between the β1 and β2 strands of p14 docks into the groove formed by the α3 helices of the roadblock domains of RagA-C (Fig. 3c and Supplementary Fig. 8). These structural features imply that the roadblock heterodimers might serve as a structural module that interacts with other related molecules via a conserved head-to-tail mode of interaction to bring about further multimerization.

These findings clearly demonstrate that p18 directly interacts with the RagA(RD)-C(RD) heterodimer via its N- and C-terminal regions through an induced fit mechanism to bind six roadblock proteins. Since p18 interacts with all other components of Ragulator and RagA(RD)-C(RD) (Supplementary Table 2) and most of the functional regions of p18 (Ser42–P161) are occupied in the final assembly (Fig. 3d and Supplementary Movie 1), it is likely that the primary function p18 is to stably anchor RagA-C onto Ragulator.

### Structural comparison between the two complexes

To examine whether the binding of RagA(RD)-C(RD) affects the structure of Ragulator, the structure of the Ragulator-RagA(RD)-C(RD) complex was superimposed on that of Ragulator. Local conformational changes occur upon assembly, specifically at the α2 and α4 helices of p18 and the β2–α2 loop of p14 (Supplementary Fig. 9). In the β2–α2 loop of p14, the binding of RagA(RD)-C(RD) induces a conformational change in Arg43 and hydrogen bond formation with Ser280 of RagA, generating a new hydrogen bond with Tyr94 of p14 (Supplementary Fig. 9a). The α2 and α4 helices of p18 undergo positional and conformational shifts to fit tighter into the grooves formed by MP1 and HBXIP, respectively (Supplementary Fig. 9b, c). The α4 helix of p18 forms new π-stacking interactions with His8 of HBXIP, and new hydrogen bonds between Asp11 and Asp80 of HBXIP (Supplementary Fig. 9c). Nevertheless, the overall structures of p18 within Ragulator and Ragulator-RagA(RD)-C(RD) align well with each other and have a similar topology (Supplementary Fig. 9d). These observations indicate that the conformational flexibility of p18 allows it to specifically wrap the subunits of Ragulator and Rag GTPase into a functional complex through an induced fit mechanism.

### p18 is essential for Ragulator-RagA-C assembly on lysosomes

To investigate the structure–function relationship between p18 and the Ragulator-Rag GTPase complex, we examined the effects of p18 mutations on the function of mTORC1 by reexpressing various p18 mutants (Supplementary Fig. 10) in p18 KO cells, which were established from p18 KO mouse embryos^17^. The function of mTORC1 was assessed by the punctate distribution of mTOR to perinuclear lysosomes (Supplementary Fig. 11). Among the mutations examined, multiple substitutions in the α2 and α4 helices (α2A and α4E) and deletion of the C-terminal 10–40 residues (CΔ10–40) caused a suppression of punctate lysosomal distribution of mTORC1. These observations suggest that these regions of p18 are important for the assembly of the Ragulator-Rag GTPase complex, which is required for mTORC1 activation on lysosomes.

**Fig. 4:**
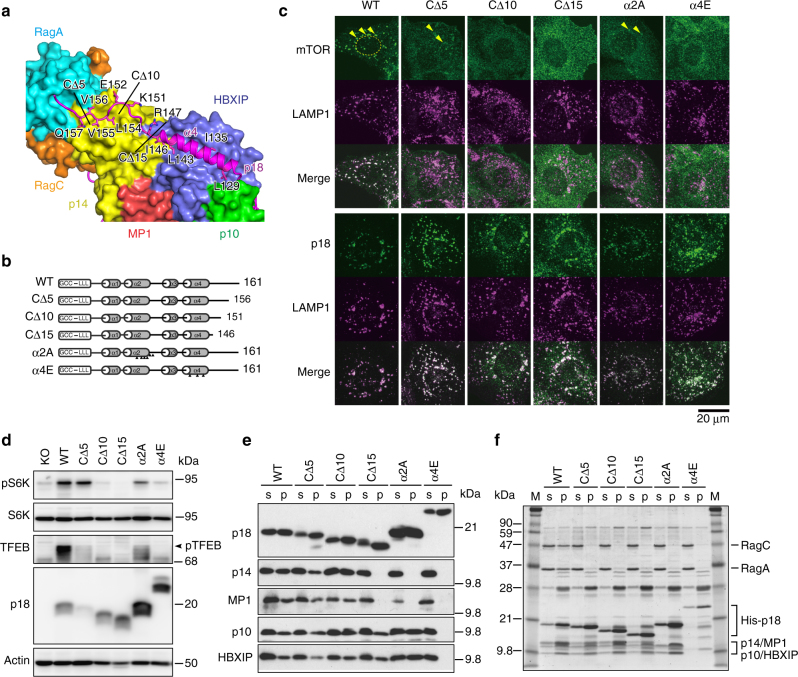
Effects of p18 mutations on the assembly and function of Ragulator-RagA-C complex. **a** Ribbon model representation of the C-terminus of p18 depicted on the molecular surface of HBXIP, p14, p10, RagA(RD), and RagC(RD). Mutated amino acids and deleted regions tested in cellular and in vitro experiments (CΔ5, CΔ10, and CΔ15) are indicated. **b** Schematic structures of p18 mutants. Arrowheads indicate the locations of amino acid substitutions. **c** p18 KO cells (KO) were transfected with wild-type p18 (WT), C-terminal deletion mutants (CΔ5, CΔ10, and CΔ15), and mutants with multiple substitutions in the α2 helix (α2A) and the α4 helix (α4E), and subjected to immunostaining for mTOR, p18, and LAMP1. Arrowheads indicate the locations of lysosomal puncta. Dotted lines denote nuclei. **d** Total cell lysates prepared from the above cells were subjected to western blotting for the indicated proteins. **e** *E. coli* expressing p14, MP1, p10, HBXIP, and the indicated mutants of His-tagged p18 were lysed, and complexes were precipitated with HisTrap beads. Soluble lysates (s) and precipitates (p) were subjected to western blotting for the indicated proteins. **f** Ragulator was purified from the above precipitates and mixed with the purified RagA-C complex, and then reprecipitated with HisTrap beads. Soluble lysates (s) and precipitates (p) were subjected to SDS-PAGE, followed by Coomassie Brilliant Blue (CBB) staining

To verify the roles of these regions, we conducted detailed analysis of CΔ5, CΔ10, CΔ15, α2A, and α4E mutants (Fig. 4a, b). Immunofluorescence analysis revealed that mTOR was delocalized from LAMP1-positive lysosomes in cells expressing CΔ10, CΔ15, and α4E mutants (critical mutant cells), although subpopulations were detectable at lysosomes in cells expressing CΔ5 and α2A mutants (moderate mutant cells) (Fig. 4c). Consistently, the activity of mTORC1 toward S6 kinase and TFEB was dramatically reduced in critical mutant cells, while moderate mutant cells retained substantial activity (Fig. 4d and Supplementary Fig. 12a).

We next examined the effects of p18 mutations on the nutrient-dependent activity of mTORC1 (Supplementary Fig. 13). Although insulin-dependent activation of S6 kinase phosphorylation was observed even in p18 KO cells, the levels of activation in critical mutant cells were almost comparable with those in p18 KO cells (Supplementary Fig. 13a). By contrast, amino acid-dependent S6 kinase phosphorylation was exclusively dependent on the presence of functional p18, and it was not activated in critical mutant cells or p18 KO cells, while moderate mutant cells showed some activation. Furthermore, TFEB phosphorylation, which was more strictly dependent on amino acids, even in WT cells, was not activated by amino acids in critical mutant cells. Time-course analyses of amino acid-dependent activities toward S6 kinase and TFEB confirmed that both activities were indeed activated in an amino acid-dependent manner, although phosphorylation of S6 kinase occurred transiently due to some negative feedback mechanisms (Supplementary Fig. 13b). These findings demonstrate that the C-terminal tail and α4 helix of p18 are crucial for amino acid-dependent activation of mTORC1.

The roles of the critical regions of p18 in the assembly of the Ragulator-Rag GTPase complex were confirmed by in vitro reconstitution analyses. Pull-down assays of recombinant proteins expressed in *E. coli* revealed that the C-terminal 10 residues (Glu152–Pro161) of p18 are dispensable for the assembly of Ragulator (Fig. 4e), but they are critical for stably anchoring RagA-C on Ragulator (Fig. 4f). The additional five residues at the C-terminus (Arg147–Lys151) are required for the binding of p14-MP1 (Fig. 4e). The α2 helix of p18 can contribute to the assembly of p14-MP1, and helix α4 is essential for the total assembly of Ragulator (Fig. 4e). These results demonstrate that the C-terminal tail and α2/α4 helices of p18 contribute to a stable assembly of the Ragulator-Rag GTPase complex.

**Fig. 5:**
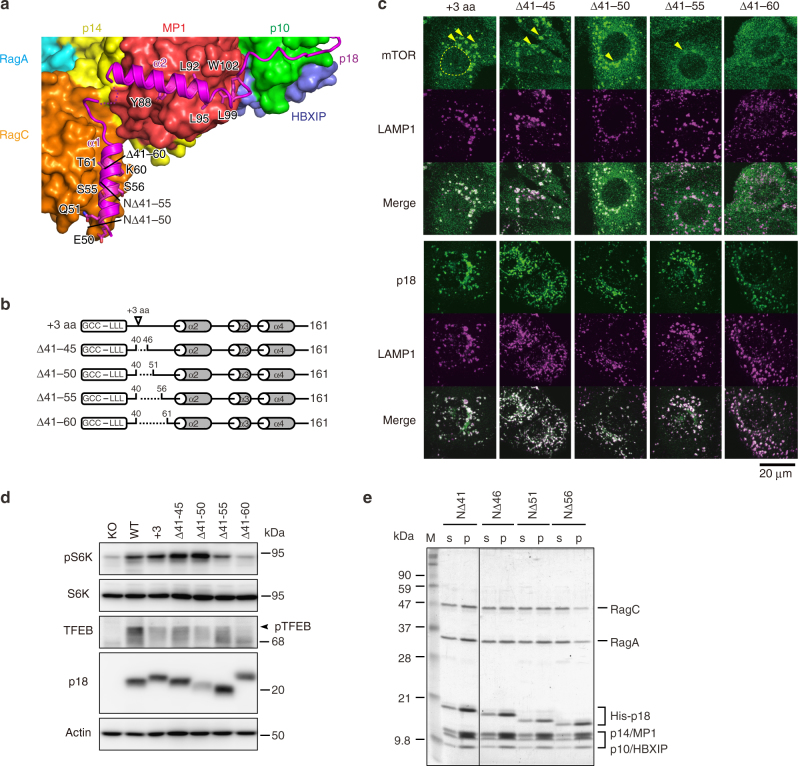
Mutational analysis of the N-terminal region of p18. **a** Ribbon models of α1 and α2 helices of p18 depicted on the molecular surface of RagC(RD) and MP1. Deletion boundaries tested in the cellular and in vitro experiments (NΔ50, NΔ55, and NΔ60) are indicated. **b** Schematic structures of N-terminal deletion mutants of p18. **c** p18 KO cells (KO) were transfected with the indicated mutants of p18, and subjected to immunostaining for p18, mTOR, and LAMP1. Arrowheads indicate the locations of lysosomal puncta. Dotted lines denote nuclei. **d** Total cell lysates prepared from the above cells were subjected to western blotting for the indicated proteins. **e** *E. coli* expressing p14, MP1, p10, HBXIP, and the indicated mutants of His-tagged p18 were lysed, and complexes were precipitated with HisTrap beads. Complexes were eluted from precipitates and mixed with the purified RagA-C complex, and then reprecipitated with HisTrap beads. Soluble lysates (s) and precipitates (p) were subjected to SDS-PAGE, followed by CBB staining

We also analyzed the contribution of N-terminal regions of p18 to the regulation of mTORC1. Cellular and in vitro reconstitution analyses showed that the N-terminal 10 residues (His41–Glu50), which are disordered in the structure of the Ragulator-RagA(RD)-C(RD) complex, are dispensable for mTOR activation, while the following region (Gln51–Lys60) in the N-terminal α1 helix (Glu50–Asn64) is required for amino acid-dependent activation of mTORC1 (Fig. 5a–e and Supplementary Figs. 12b and 13a, b). These results indicate that, in addition to the C-terminal tail of p18, the N-terminal helix α1 is also crucial for stabilizing the interaction between RagA-C and Ragulator, which is required for amino acid-dependent activation of mTORC1. Taken together, the functional and structural information suggest that full assembly of the Ragulator-Rag GTPase complex via p18 is required to ensure the amino acid-dependent regulation of mTORC1 activity on lysosomes.

## Discussion

**Fig. 6:**
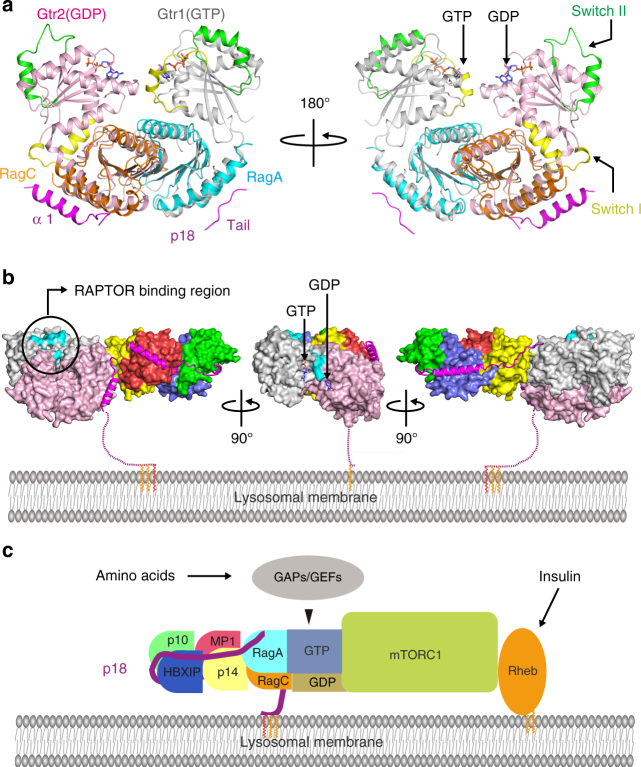
Modeling of the functions of the Ragulator-Rag GTPase complex on lysosomes. **a** Ribbon model representation of the RagA(RD)-C(RD) structure superimposed on that of the Gtr1p-2p complex (left panel). The structure is viewed from different angles rotated at 180° along the vertical axis (right panel). The locations of switch I and II regions are indicated on the Gtr1p-2p complex, and are shown in yellow and green, respectively. **b** Model of the complete Ragulator-RagA-C complex anchored on the lysosomal membrane. p18 is shown in stick and ribbon representation on the molecular surface of the simulated Ragulator-Rag GTPase complex. The model is viewed from differential angles rotated at 90° along the vertical axis. The RAPTOR-binding region and GTP/GDP-binding sites are indicated. **c** Schematic model of mTORC1 activation by the Ragulator-Rag GTPase complex on lysosomes

In this study, we determined crystal structures of Ragulator and the Ragulator-RagA(RD)-C(RD) complex, which are composed of roadblock heterodimers wrapped in an orderly fashion by p18. During purification of Ragulator, we noticed the presence of a pre-Ragulator complex (p18-p10-HBXIP; Supplementary Fig. 2d). Furthermore, in vitro reconstitution assays in *E. coli* show that p18 is able to form a complex with p10-HBXIP alone, but p14-MP1 requires p10-HBXIP for complex formation with p18, and p18 is required for the assembly of p14-MP1 and p10-HBXIP (Supplementary Fig. 14). These findings suggest a stepwise assembly mechanism for Ragulator formation, in which p18 first specifically captures p10-HBXIP through multiple interactions with its middle region, including the M-loop, α3, and α4 helices, and then assembles p14-MP1 mainly via interactions between MP1 and the N-terminal α2 helix of p18, which is consistent with a previous report that the C-terminal region of p18 is not needed for interaction with p14-MP1^30^. The assembled Ragulator then anchors Rag GTPases by clipping their roadblock domains with the C-terminal tail and α1 helix of p18 (Figs. 3d and 6a). Through this stepwise process, the Ragulator-Rag GTPase complex is precisely assembled on the surface of lysosomes.

To assess the complete structure of the Ragulator-RagA-C complex containing the GTP-binding domains, we superimposed the previously determined structure of the Gtr1p–Gtr2p complex^29^ onto that of human RagA(RD)-C(RD) in our structure (Fig. 6a). The superimposition suggests that the full Ragulator-RagA-C complex lying on the surface of the lysosomal membrane has its RAPTOR-binding region^31^ (RagA nucleotide-binding domain) located on the side of the molecule opposite to the lysosomal membrane (Fig. 6b). This orientation may be advantageous to capture mTORC1 via RAPTOR on the surface of the lysosomal membrane and allows close contact with RHEB, which directly activates mTORC1. The structure also reveals that the C-terminal tail of p18 does not reach the nucleotide-binding domain of RagA, and that the N-terminal domain does not interact with the nucleotide-binding domain of RagC (Fig. 6a). Furthermore, structural comparison between RagA(RD)-C(RD) and the roadblock domains of Gtr1p–Gtr2p indicates that the binding of Ragulator does not induce a dramatic conformational change in RagA(RD)-C(RD). These in vitro observations suggest that the binding of Ragulator may not directly affect the activity of RagA-C, although it is likely that Ragulator requires additional factors to regulate Rag GTPases in vivo.

Analysis of the structure–function relationship between p18 and the Ragulator-Rag GTPase complex suggests that the C-terminal tail and the N-terminal α1 helix of p18 are crucial for amino acid-dependent activation of mTORC1. Since these regions are required for holding RagA-C onto Ragulator, p18-mediated full assembly of the Ragulator-Rag GTPase complex may be a prerequisite for amino acid-dependent regulation of mTORC1. In this study, we also found that insulin-dependent activity of mTORC1 toward S6 kinase was retained to some extent even in p18 KO cells, while mTORC1 activity toward TFEB was largely dependent on amino acids and substantially attenuated by the loss of p18 function. These results underscore the crucial role of the Ragulator-Rag GTPase complex in amino acid-dependent regulation of mTORC1, and suggest that the insulin pathway might activate S6 kinase independently of the Ragulator-Rag GTPase complex on lysosomes.

In the structure of the Ragulator-RagA-C complex, the roadblock domains [α1–β1–β2–α2–β3–β4–β5–(α3)], which were originally identified in the *Drosophila roadblock* gene that encodes a dynein-associated protein^22^, are present in all components except p18. This domain is also shared in several small GTPase-related molecules and functions as a platform for small GTPase signaling^21^. Furthermore, our structure shows that the three pairs of roadblock domains assemble via head-to-tail interactions, forming a unique molecular surface that may facilitate further interactions with additional molecules that have related-modules, such as roadblock and/or longin domains^21,32^. Therefore, it is possible that p18 functions to create a platform for regulators of Rag GTPase, such as GEFs^25^ and GAPs (Fig. 6c). Since several known regulators of Rag GTPases, including GATOR1^33^, FLCN^34^, and c17orf57^35^, contain longin-related domains, these factors may specifically interact with the Ragulator-Rag GTPase platform to regulate the activity of Rag GTPases on lysosomal membranes.

Recently, a crystal structure of the Ego1–Ego2–Ego3 ternary complex (Ego-TC), a yeast ortholog of Ragulator, was reported^36^. The Ego-TC structure differs from that of human Ragulator, consisting of a heterotrimer of Ego2, Ego3, and a C-terminal short region of Ego1 (Leu146–Phe184). However, the mode of interaction between Ego1 and Ego2-3 is somewhat similar to that between p18 and HBXIP-p14; the C-terminal α-helix and tail region of Ego1 and p18 interact with the surface of Ego2-3 and HBXIP-p14, respectively. Furthermore, the structure of Ego-TC was aligned with that of the p18-HBXIP-p14 complex in our structure with an overall root mean square deviation of 2.14 Å for all Cα atoms of across both components (Supplementary Fig. 15). Although the interaction sites between Ego-TC and Gtr1p–Gtr2p GTPases remain unknown, these findings suggest that the function of Ego1/p18 may be, at least in part, conserved during evolution. Since the Ego-TC complex was shown to be crucial for promoting Rag GTPase-dependent TORC1 signaling in yeast^36^, comparative analysis of the overall structures of the Ragulator-Rag GTPase complex in human beings and yeast will likely prove informative for understanding the basic mechanisms of its regulation.

During the review process of this paper, de Araujo et al. reported crystal structures of human Ragulator and Ragulator in complex with the roadblock domains of RagA-C^37^. The overall views of their structures are basically identical to ours, although there are some minor differences in the N- and C-terminus of each component and M-loop region of p18. Particularly, the critical interaction between RagA(RD) and the C-terminal tail of p18 is significantly perturbed in their structure, potentially due to extra amino acids at the N-terminus of p14 (Supplementary Table 3 and Supplementary Fig. 16). These structural features together with our data on the structure–function relationship underscore the crucial role of p18-mediated assembly of the Ragulator-Rag GTPase complex as a key regulatory platform for amino acid-dependent mTORC1 signaling. Identification of the critical regulatory sites on the surface of this complex may provide new chemotherapeutic targets for small molecules to treat human diseases, such as cancer and diabetes mellitus, which are associated with the dysregulation of mTORC1 signaling.

## Methods

### Expression and purification of Ragulator and RagA-C

To increase the solubility of the p18 protein in *E. coli*, substitution of Ser98 to Asp, which mimics the potential phosphorylation at this site, was introduced, although this substitution did not affect the function of Ragulator (Supplementary Figs. 2c and 11). The mutated cDNA fragment of the C-terminal region of human p18 (Ser42–Pro161) was cloned into the *Eco*RI-*Not*I sites of the pETDuet1 vector (Novagen), in which a tobacco etch virus (TEV) protease recognition site was introduced. PCR primers used in this study are listed in Supplementary Table 4. Full-length cDNAs of MP1 (Ala2–Ser124) and p14 (Met1–Ser125) were cloned into the *Nco*I-*Hind*III site and the *Nde*I-*Xho*I sites of the pACYCDuet1 vector (Novagen), respectively. The C-terminal half of HBXIP (Glu83–Ser173), which is a well-conserved short form, and the full-length cDNA of p10 (Met1–Val99) were cloned into the *Nco*I-*Hind*III site and the *Nde*I-*Xho*I sites of the pRSFDuet1 vector (Novagen), respectively. Expression vectors were simultaneously transfected into *E. coli* BL21(DE3) cells (Novagen), and the cells were cultured in the presence of the recommended amounts of ampicillin, kanamycin, and chloramphenicol in 2-l flasks at 37 °C. Protein expression was induced by 0.5 mM isopropyl-β-D-1-thiogalactoside (IPTG) at 30 °C for 1.5 h, and cells were collected by centrifugation and stored at −30 °C until needed. Cells were lysed in 50 ml of Lysis buffer consisting of 20 mM Tris-HCl (pH 8.0), 0.5 M NaCl, 40 mM imidazole, 0.2% Nonidet-P40 (NP-40), 1 mM phenylmethylsulfonyl fluoride (PMSF), and 1 mg/ml lysozyme. Lysates were sonicated five times for 1 min each time, and centrifuged at 30,000 × *g* for 10 min. Protein was purified from the cleared supernatant using a HisTrap HR column (5ml) with a linear gradient of 40–200 mM imidazole, followed by a Superdex 200 Increase 10/300GL column (GE Healthcare). Peak fractions containing the eluted protein were digested with Ac TEV protease (Invitrogen) at 4 °C for 7 h. After passing the TEV protease reaction through a HisTrap HR column, Ragulator was purified by MonoQ 10/100 GL column chromatography with a linear gradient of 0.1–0.35 M NaCl.

For expression of full-length human RagA and mouse RagC, the appropriate cDNAs were cloned into the *Bam*HI-*Not*I sites and the *Bgl*II-*Xho*I sites of the pETDuet1 vector, respectively. The resultant constructs were transformed into *E. coli* BL21 (DE3) cells, and cells were cultured in the presence of ampicillin in 2-l flasks at 37 °C. Protein expression was induced by the addition of 0.5 mM IPTG, and culturing continued at 30 °C for 1.5 h. Cells were collected by centrifugation and lysed in the Lysis buffer consisting of 20 mM Tris-HCl (pH 8.0), 0.5 M NaCl, 40 mM imidazole, 0.2% NP-40, 5% glycerol, 5 mM MgCl_2_, 10 µM GTP/GDP, 4 mM β-mercaptoethanol, 1 mM PMSF, and 1 mg/ml lysozyme. Protein was purified from the cleared lysates using a HisTrap HR column (5 ml) followed by a Superdex 200 Increase 10/300GL column, and peak fractions containing the target protein were digested with Ac TEV protease at 4 °C for 7 h. After passing the reaction through a HisTrap column, the RagA/C complex was purified by HiTrap Q HR (5 ml) column chromatography with a linear gradient of 0.1–0.4 M NaCl. To form the heteroheptamer, purified Ragulator and RagA/C were mixed and subjected to gel filtration on a Superdex 200 increase 10/300GL column.

For coexpression of Ragulator with the roadblock domains (RD) of RagA-C, RagA(RD) consisting of residues Asn183–Arg313 and RagC(RD) consisting of residues Gln238–Ser375 were cloned into the *Bam*HI-*Not*I sites and the *Nde*I-*Xho*I sites in the pETDuet1 vector, respectively, and the resulting *BamH*1-*Xho*I fragment was then cloned into the *Bgl*II-*Xho*I sites in the pETDuet1 vector harboring the p18 gene. The vector was transformed along with pACYCDuet1-MP1/p14 and pRSFDuet1-p10/HBXIP into *E. coli* BL21(DE3) cells, and cells were cultured in the presence of ampicillin, kanamycin, and chloramphenicol in 2-l flasks at 37 °C. Protein expression was induced by the addition of 0.5 mM IPTG, and culturing continued at 20 °C overnight. Cells were collected by centrifugation and lysed in 50 ml of Lysis buffer consisting of 20 mM Tris-HCl (pH 8.0), 0.5 M NaCl, 50 mM imidazole, 0.2% NP-40, 5% glycerol, 4 mM β-mercaptoethanol, 1 mM PMSF, and 1 mg/ml lysozyme. Protein was purified from cleared lysates using a HisTrap HR column (5 ml) with a linear gradient of 50–200 mM imidazole, followed by a Superdex 200 Increase 10/300GL column. Peak fractions containing the target protein were digested with Ac TEV protease at 4 °C for 7 h. After passing the TEV protease reaction through a HisTrap column, the Ragulator-RagA(RD)-C(RD) complex was purified by a second passage through the Superdex 200 Increase 10/300GL column.

### Crystallization

Purified proteins were concentrated to ~10 mg/ml for Ragulator and ~12 mg/ml for the Ragulator-RagA(RD)-C(RD) complex, and crystallization screening was performed with a TTP Labtech Mosquito crystallization robot by sitting drop vapor diffusion at 20 °C. After optimization of the crystallization conditions, the best crystals were grown from 100 mM Tris-HCl (pH 9.0), 20 mM alcohol mix (2-propanol, 1,6-hexanediol, 1,4-butanediol, 1-butanol, and 1,3-propanediol), 13.75% PEG1000, 13.75% PEG3350, and 13.75% 2-methyl-2,4-pentanediol (MPD) for Ragulator, and 100 mM sodium citrate (pH 6.0), 200 mM ammonium acetate, and 15% PEG4000 for Ragulator-RagA(RD)-C(RD). Crystals were flash-frozen in liquid nitrogen after cryoprotection in mother liquor supplemented with 15% PEG1000, 15% PEG3350, and 15% MPD for Ragulator, and 30% glycerol for Ragulator-RagA(RD)-C(RD).

### Data collection and structure determination

All diffraction data sets were collected on BL44XU^38^ at SPring-8, and indexed, integrated, and scaled using HKL2000^39^, autoPROC^40^ with XDS^41^, AIMLESS^42^, and POINTLESS^43^. Structures were determined by molecular replacement using Phaser^44^ within the CCP4 suite^45^, with the previously reported crystal structures of p14-MP1^27^ and HBXIP^46^ (PDB: 1VET and 3MSH, respectively) as templates for Ragulator, and the Ragulator and roadblock domains of Gtr1p-2p^29^ (PDB: 4ARZ) as templates for Ragulator-RagA(RD)-C(RD). All models were refined by Refmac5^47^, PHENIX^48^, and BUSTER^49^, with manual revision using Coot^50^. Hydrophobicity was calculated according to the Eisenberg hydrophobicity scale^51^. X-ray diffraction and refinement statistics are summarized in Supplementary Table 1. Analysis of interacting interfaces was performed using PDBePISA^52^. All figures were made with the PyMOL Molecular Graphics System, Version 1.7 (Schrödinger, LLC).

### Cell culture and transfection

p18 KO cells were established from p18 KO mouse embryos by immortalizing with SV40 large T antigen^17^, and cultured at 37°C in a humidified atmosphere containing 5% CO_2_ in Dulbecco’s Modified Eagle’s Medium (DMEM) supplemented with 10% (v/v) fetal bovine serum (FBS) and penicillin/streptomycin. Gene-transfer experiments were carried out using PiggyBac transposon vector system (System Biosciences). To analyze the amino acid- and insulin-dependent activity of mTORC1, semiconfluent cell cultures were washed with PBS and then incubated in DMEM (high glucose) with or without amino acids for 1 h. Insulin (10 μg/ml) was directly added to the medium. For time-course analyses, cultured cells were pretreated for 1 h with amino acid-free DMEM (high glucose), and were further incubated in DMEM (high glucose) with amino acids for each period.

### Western blotting

Cells were lysed in ODG buffer (20 mM Tris-HCl, pH 7.4, 0.15 M NaCl, 1 mM EDTA, 1% NP-40, 2% octyl-β-D-glucoside, 5% glycerol, 1 mM Na_3_VO_4_, 10 mM NaF, and protease inhibitors). Equal amounts of total proteins were separated by SDS-PAGE and transferred onto polyvinylidene difluoride (PVDF) membranes. Membranes were blocked and incubated with primary antibodies, followed by incubation with HRP-conjugated secondary antibodies. Signals were visualized on a WSE6200H LuminoGraph II (ATTO, Tokyo, Japan). Representative blots obtained from at least three independent experiments are shown. For pull-down assays in *E. coli*, cell lysates expressing His-tagged p18 were incubated with HisTrap beads, and precipitates were subjected to Coomassie Brilliant Blue staining and western blotting. The following antibodies were used: anti-p18, anti-p14, anti-MP1, anti-HBXIP, anti-p10, anti-mTOR, and anti-phospho-S6K (Cell Signaling Technology), anti-actin, anti-LAMP1 and anti-S6K (Santa Cruz Biotechnology), and anti-TFEB (Bethyl Laboratories Inc). Catolog numbers and dilutions of these antibodies are listed in Supplementary Table 5. All experiments were repeated at least three times, and representative blots are displayed in all figures. Uncropped images of western blots are shown in Supplementary Figs. 17–20.

### Immunofluorescence

Cells were cultured on coverslips and fixed with 1% paraformaldehyde in PBS at room temperature for 10 min. For detection of HBXIP, fixed specimens were incubated in methanol at room temperature for 10 min, and then treated with 50 μg/ml digitonin in PBS (digitonin-PBS) at room temperature for 10 min for permeabilization. After 1 h of blocking with Blocking-One (Nacalai Tesque, Kyoto, Japan), specimens were incubated with primary antibodies at room temperature for 3 h, washed three times with digitonin-PBS, incubated with Alexa488 or Alexa594-labeled secondary antibodies at room temperature for 1 h, and washed four times with digitonin-PBS. Finally, coverslips were immersed in ProLong Gold antifade reagent and mounted on glass slides. Specimens were then subjected to observation with a confocal laser-scanning microscope (FV1000, Olympus). All experiments were repeated at least three times and representative images are displayed in all figures.

### Data availability

Coordinates and structure factors have been deposited in the Protein Data Bank under accession numbers 5X6U (Ragulator) and 5X6V (Ragulator-RagA[RD]-C[RD]). Other data are available from the corresponding authors upon reasonable request.

## Electronic supplementary material

Supplementary Information

Description of Additional Supplementary Files

Supplementary Movie 1
