# Supplementary material for: Structural basis for the assembly of the Ragulator-Rag GTPase complex
Source: Nat Commun. 2017 Nov 20;8:1625. doi: 10.1038/s41467-017-01762-3 (PMC5696360; doi:10.1038/s41467-017-01762-3)
Supplement: Supplementary file 1 — Supplementary Information [file 41467_2017_1762_MOESM1_ESM.pdf]

Supplementary Figure 1

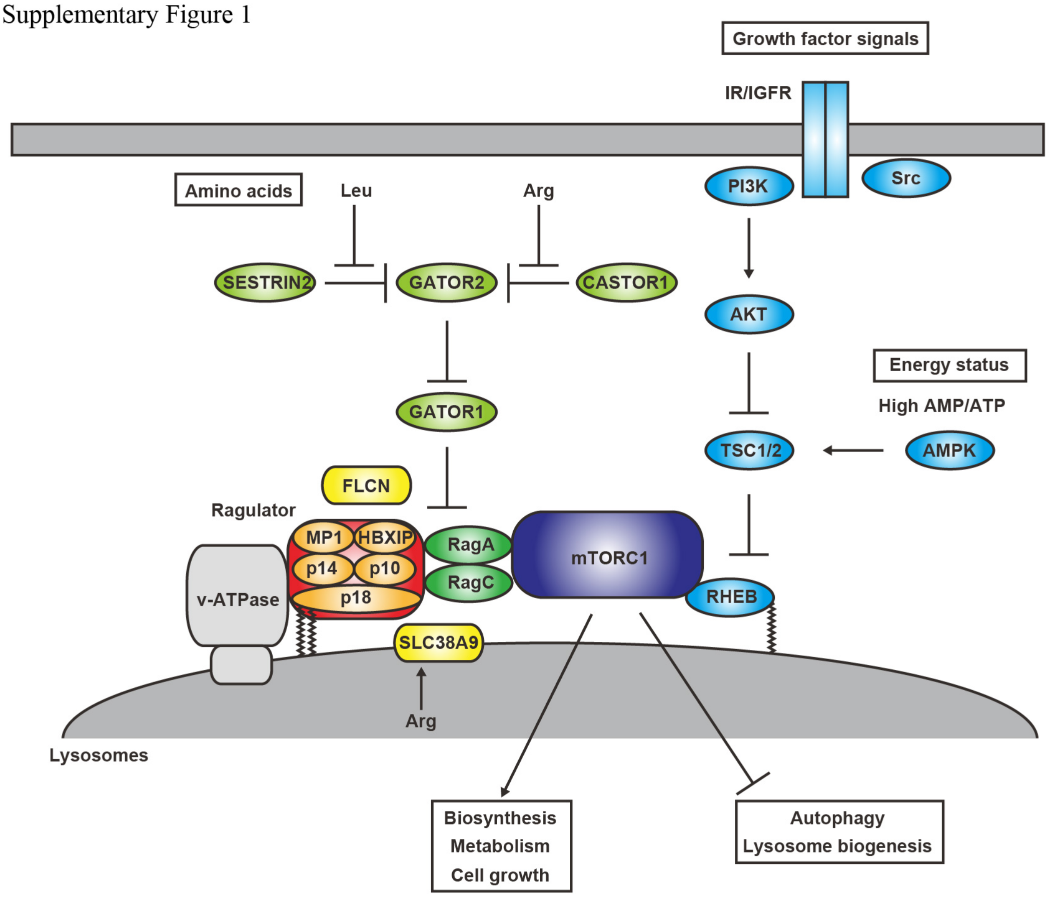

Supplementary Figure 1 | Ragulator and mTORC1 signalling.
A schematic model of Ragulator and the mTORC1 signalling pathway.

Supplementary Figure 2

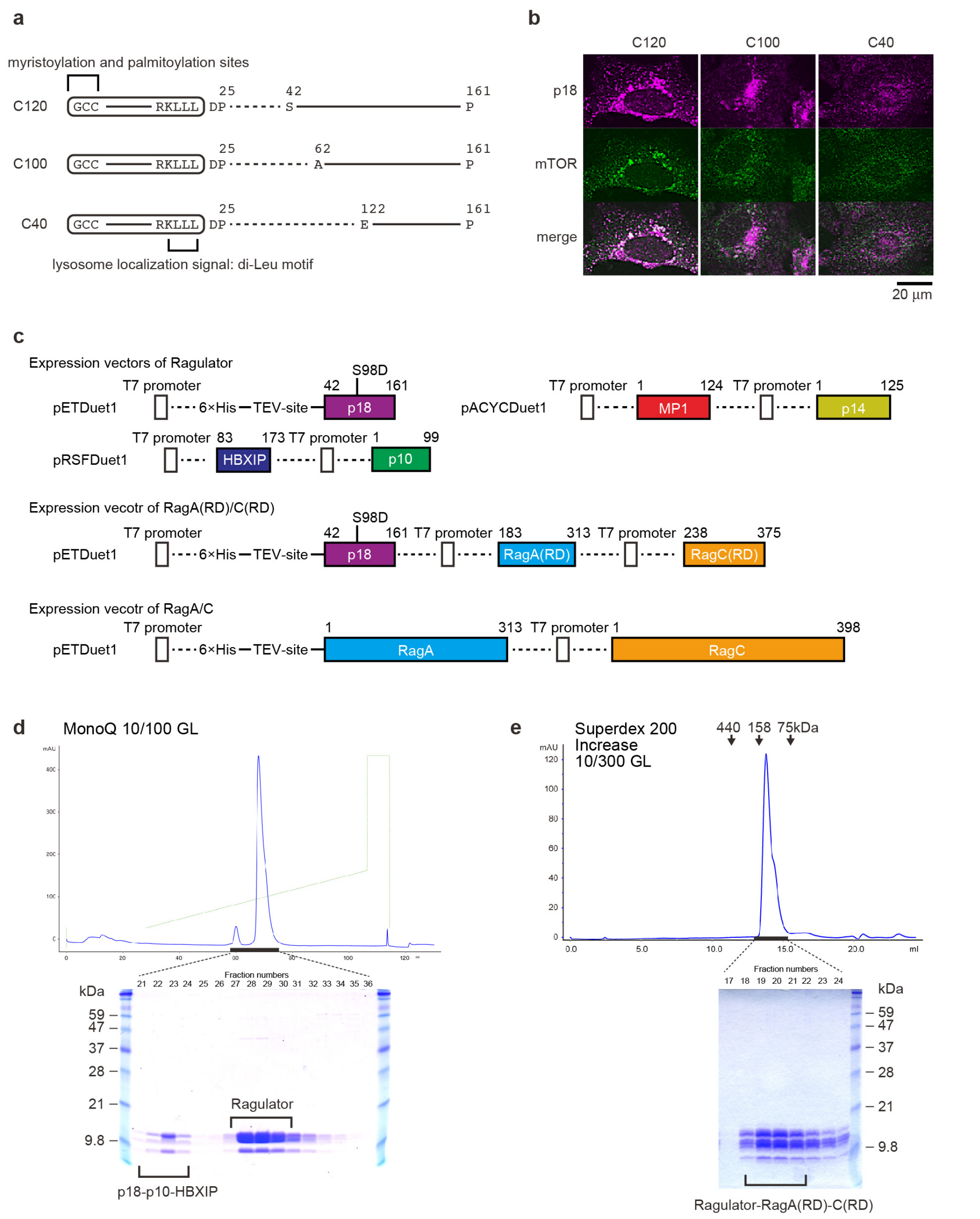

Supplementary Figure 2 | Purification of Ragulator and Ragulator- RagA(RD)-C(RD). **a,** Schematic structures of p18 mutants lacking Ser26–His41 (C120), Ser26–Thr61 (C100) and Ser26–Ser121 (C40). **b,** p18 KO cells were transfected with the above constructs, and subjected to immunostaining for p18 and mTOR. Merged images are also shown. **c,** Schematic diagrams of the constructs used for p18, MP1, p14, p10, HBXIP, RagA(RD), RagC(RD), RagA, and RagC. **d,** MonoQ10/100 column chromatogram of Ragulator (upper panel) and SDS-PAGE analysis of the indicated fractions containing proteins (lower panel). Fractions containing the p18-p10-HBXIP trimer are shown. **e,** Superdex 200 Increase 10/300GL column chromatogram of the Ragulator-RagA(RD)-C(RD) complex (upper panel), and SDS-PAGE analysis of the indicated fractions containing the complex (lower panel). Elution positions of molecular weight marker proteins (Ferritin, 440 kDa; Aldolase, 158 kDa; Conalbumin, 75 kDa) are indicated by arrows.

Supplementary Figure 3

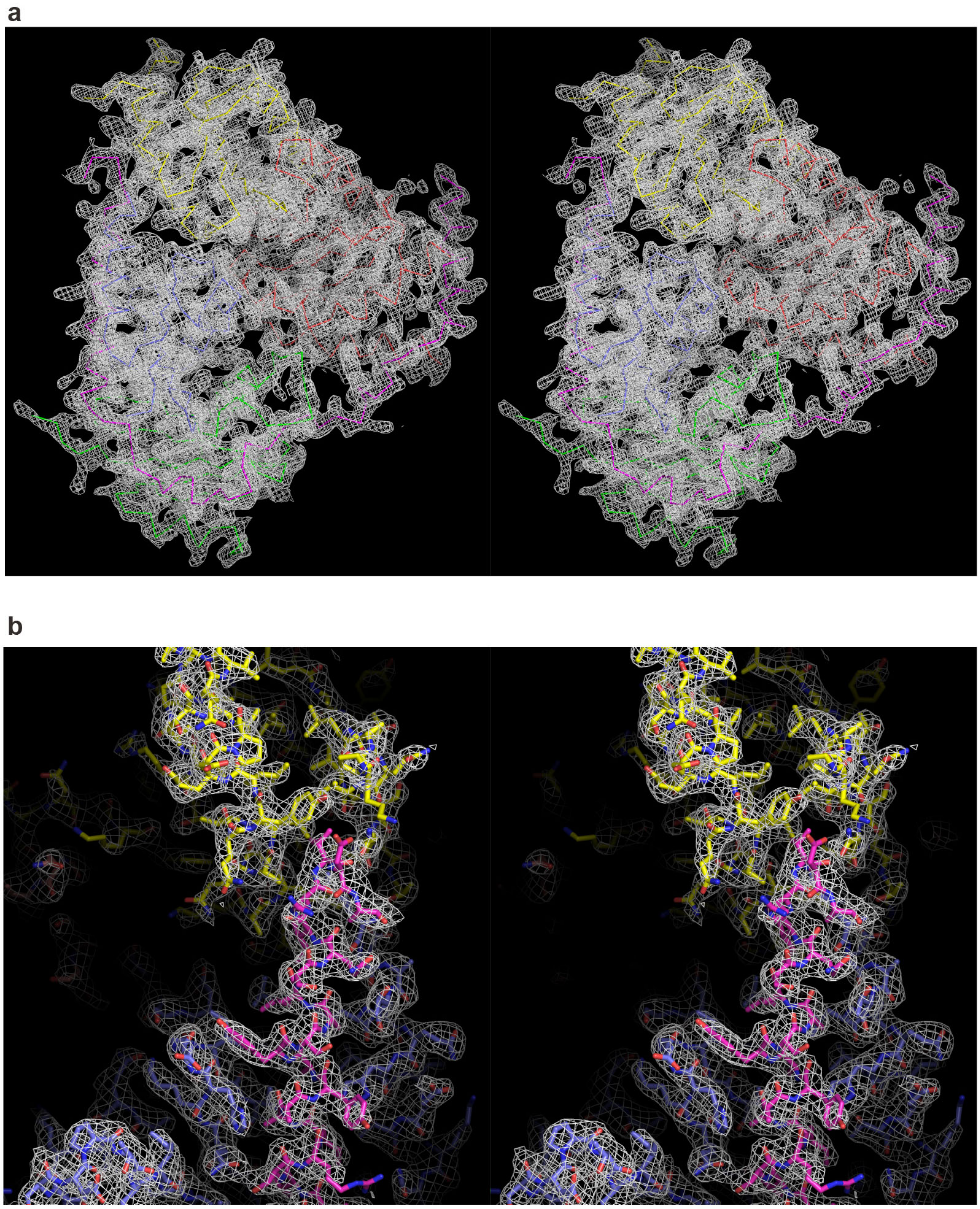

Supplementary Figure 3 | Stereo diagrams showing the 2m*F*o-D*F*c electron density map of the Ragulator at 2.40 Å resolution. a, The electron density map contoured at 1.0σ is shown in white. The Cα traces of the Ragulator in the asymmetric unit are included. **b**, Representative selection of the electron density map in the C-terminal region of p18.

Supplementary Figure 4

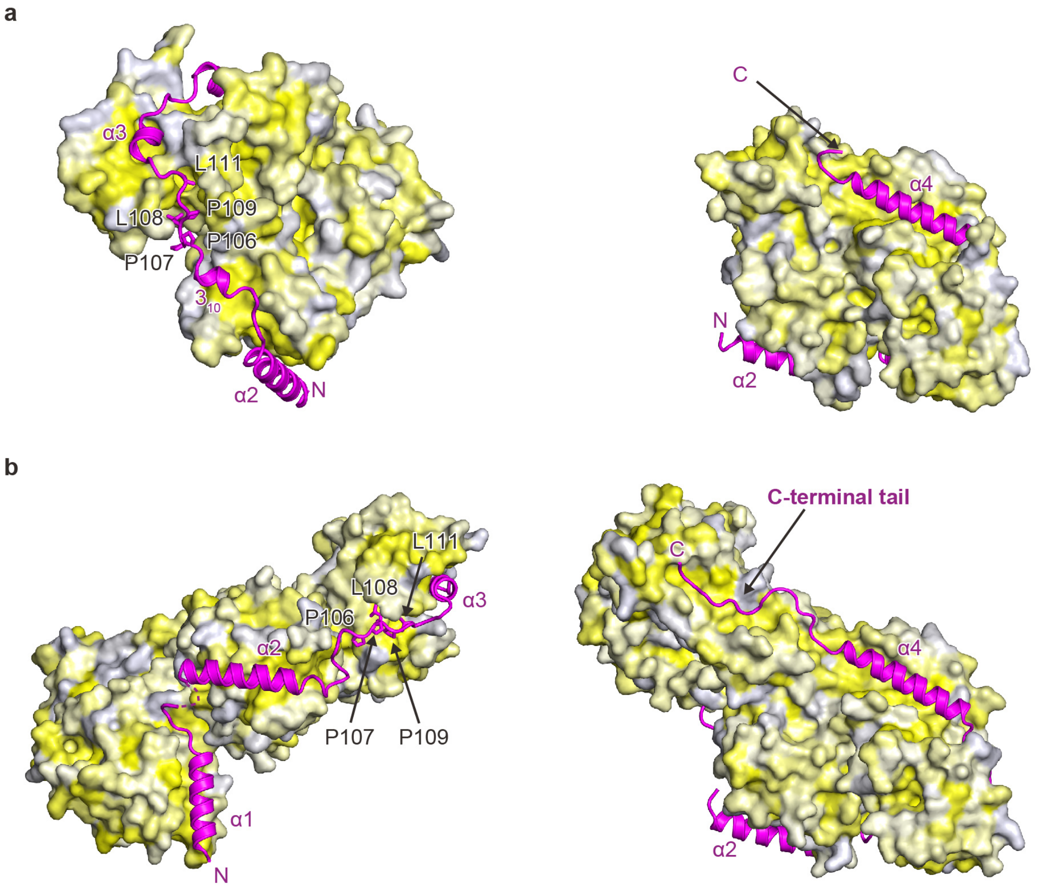

Supplementary Figure 4 | Hydrophobic interactions between p18 and other components of Ragulator and the Ragulator-RagA(RD)-C(RD) complex.

a, Surface representation of Ragulator with p18 omitted, colour-coded by hydrophobicity (yellow, hydrophobic; white, hydrophilic). Ribbon model representations of the middle part and helix α4 of p18 depicted on a hydrophobic surface representation of MP1-p10 (left panel) and p14-HBXIP (right panel).

b, Surface representation of Ragulator-RagA(RD)-C(RD) with p18 omitted, colour-coded by hydrophobicity (yellow, hydrophobic; white, hydrophilic). Ribbon models of helices α1 and α2 helices and the C-terminal tail of p18 are depicted on hydrophobic surface representations of MP1 and RagC(RD) (left panel) and HBXIP, p14, and RagA(RD) (right panel).

Supplementary Figure 5

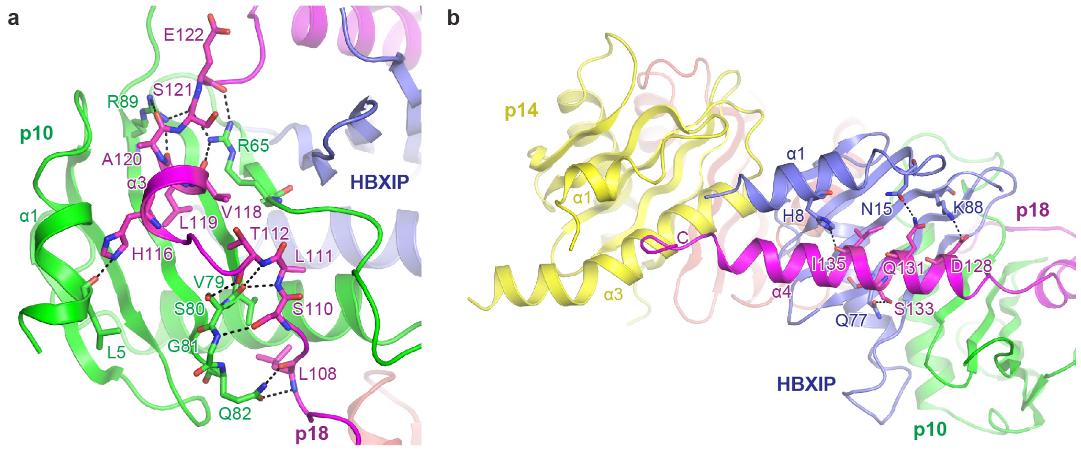

Supplementary Figure 5 | Interactions crucial for Ragulator assembly

a, Ribbon model representation of the interactions between helices α3 of p18 and p10. **b,** Ribbon model representation of the interaction between helices α4 of p18 and HBXIP.

Supplementary Figure 6

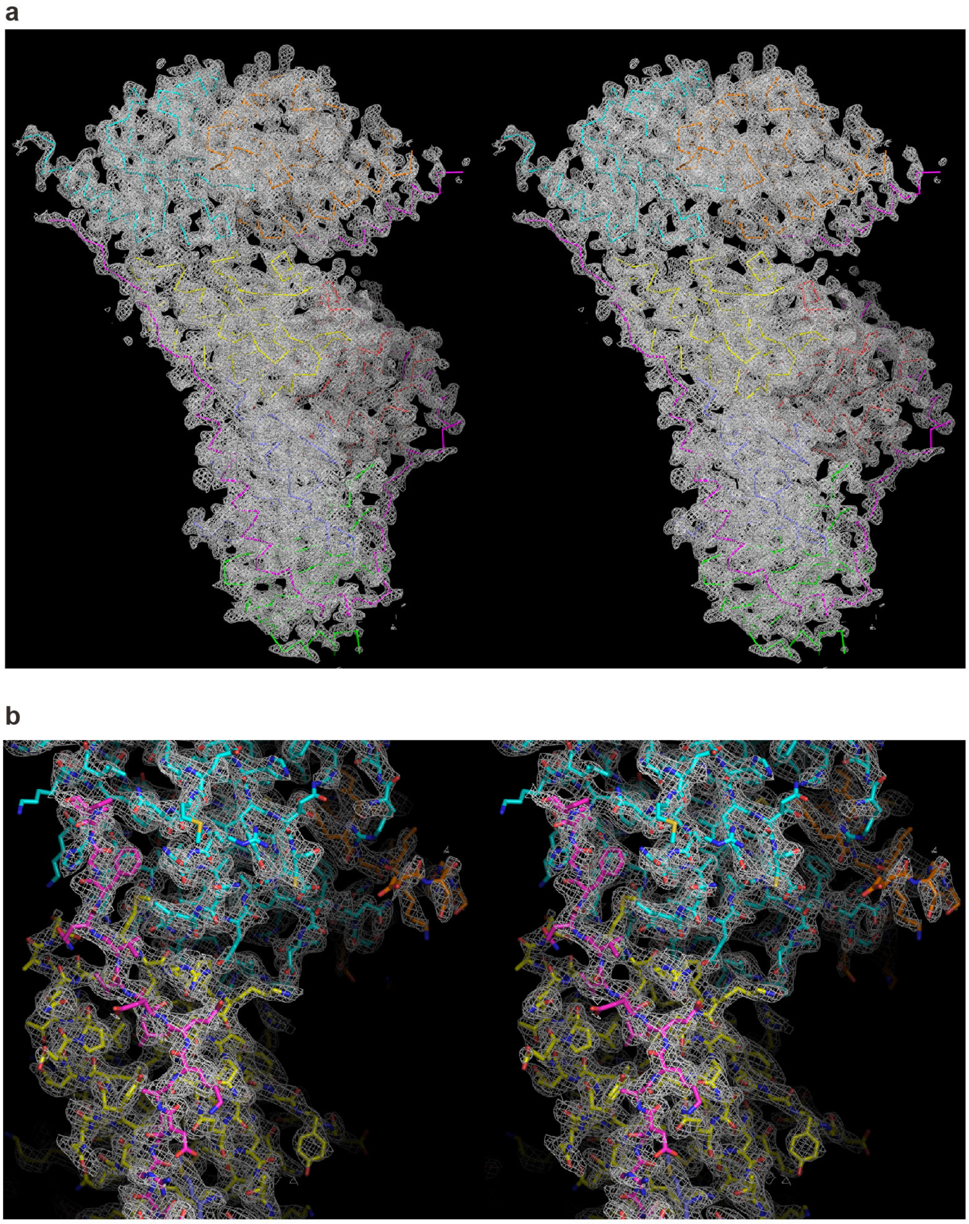

Supplementary Figure 6 | Stereo diagrams showing the 2m*F*o-D*F*c electron density map of the Ragulator-RagA(RD)-C(RD) complex at 2.02 Å resolution. a, The electron density map contoured at 1.0σ is shown in white. The Cα traces of the Ragulator-RagA(RD)-C(RD) complex in the asymmetric unit are included. **b**, Representative selection of the electron density map in the C-terminal region of p18.

Supplementary Figure 7

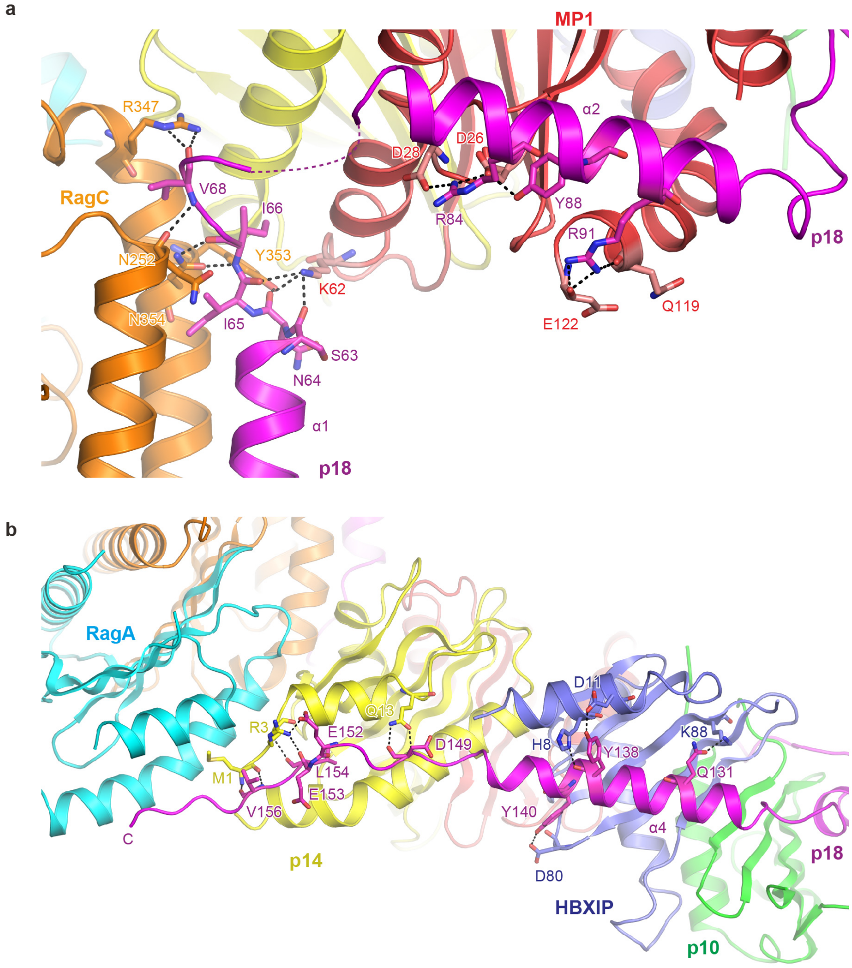

Supplementary Figure 7 | Interactions crucial for Ragulator-RagA(RD)-C(RD) assembly
a, Ribbon model representation of the interactions between helices α1 and α2 of p18 and RagC(RD) and MP1, respectively. **b,** Ribbon model representation of the interaction between the C-terminal tail of p18, p14, and RagA(RD).

Supplementary Figure 8

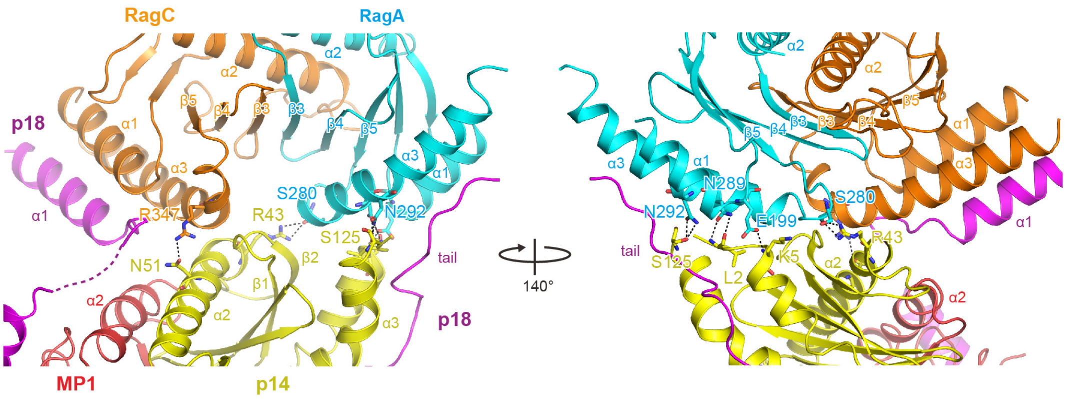

Supplementary Figure 8 | Interactions between RagA(RD)-C(RD) and p14-MP1 roadblock dimers. Ribbon model representation of interactions between RagA(RD)-C(RD) and p14-MP1 roadblock dimers (left panel). The structure is viewed from different angles rotated 140° along the vertical axis (right panel). The α1 helix and C-terminal tail of p18 capture RagC(RD) and RagA(RD), respectively.

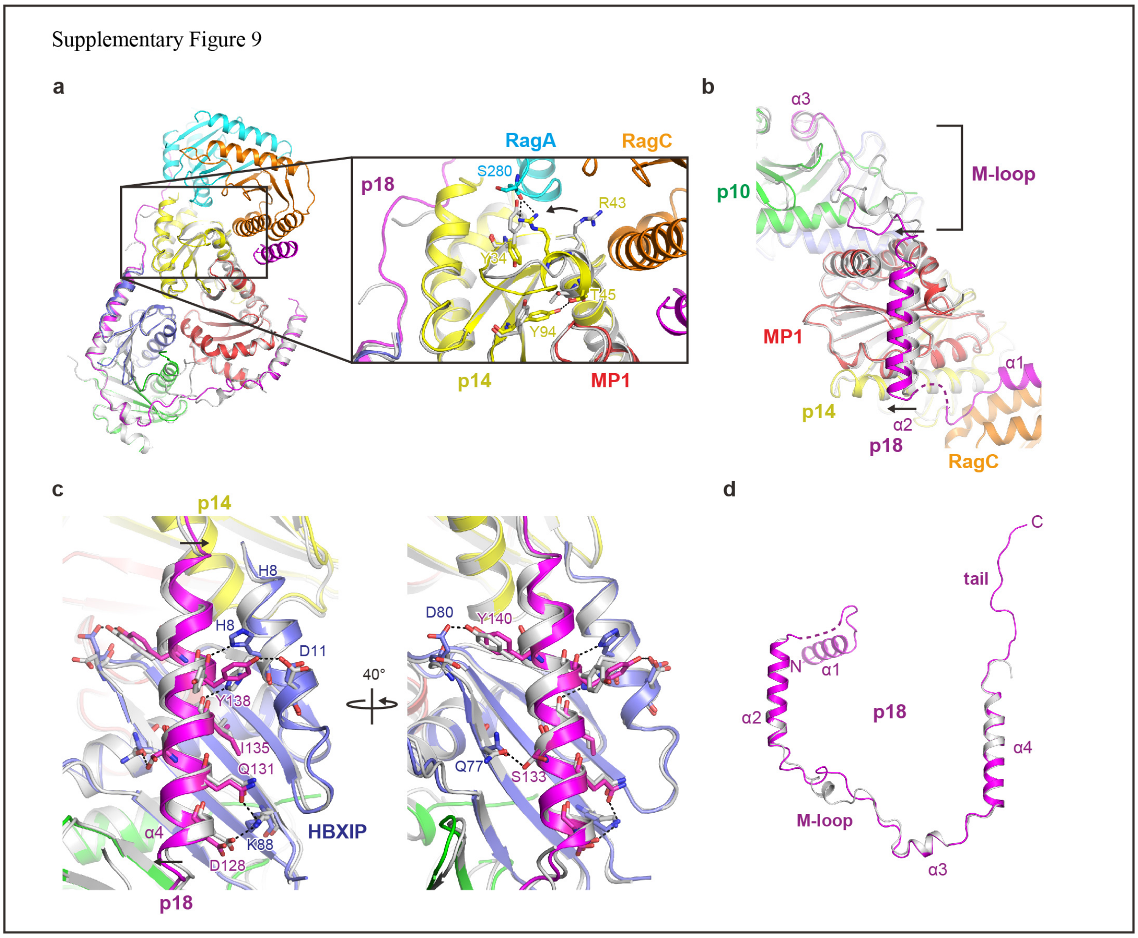

Supplementary Figure 9 | Structural comparison between Ragulator and Ragulator-RagA(RD)-C(RD).
a, Ribbon model of Ragulator superimposed on that of the Ragulator-RagA(RD)-C(RD) complex. Ragulator is shown in grey. A magnified view of the interaction site between p14 and RagA(RD) is boxed. **b,** Structural changes in helix α2 and the M-loop of p18 upon Ragulator-RagA(RD)-C(RD) complex formation. **c,** Structural changes in helix α4 of p18 upon Ragulator-RagA(RD)-C(RD) complex formation (left panel). The structure is viewed from different angles rotated 40° along the vertical axis (right panel). **d,** Ribbon model representation of p18 in Ragulator superimposed on that of the Ragulator-RagA(RD)-C(RD) complex.

Supplementary Figure 10

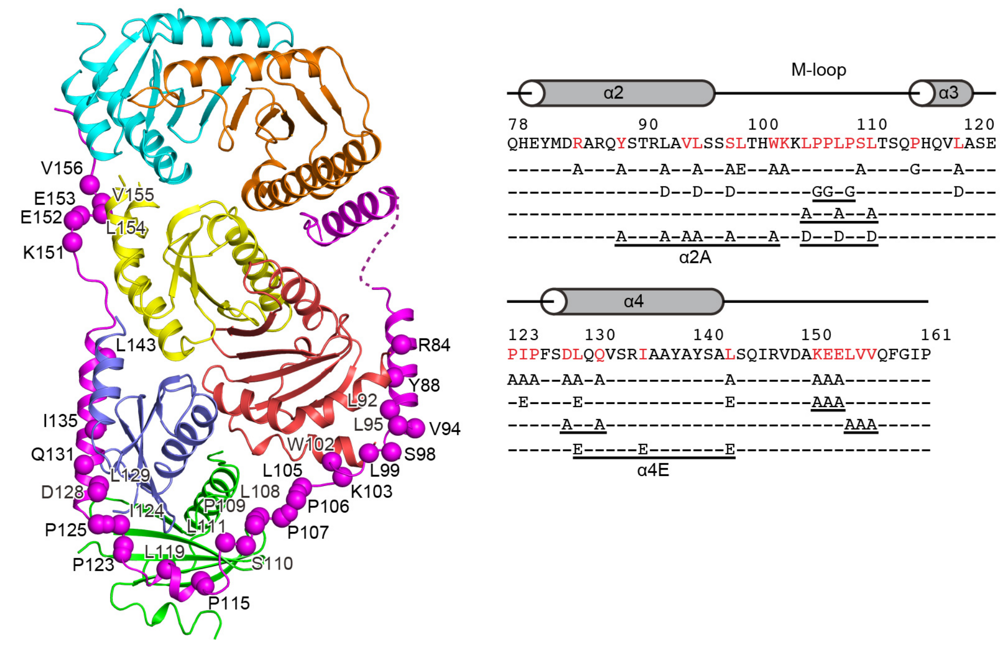

Supplementary Figure 10 | Effects of p18 mutations on the function of mTORC1.
Amino acids substituted in p18 mutants are shown as spheres in the ribbon model of the Ragulator-RagA(RD)-C(RD) complex (left panel) and in the primary structure of p18 (right panel). Multiple substitutions are underlined.

Supplementary Figure 11

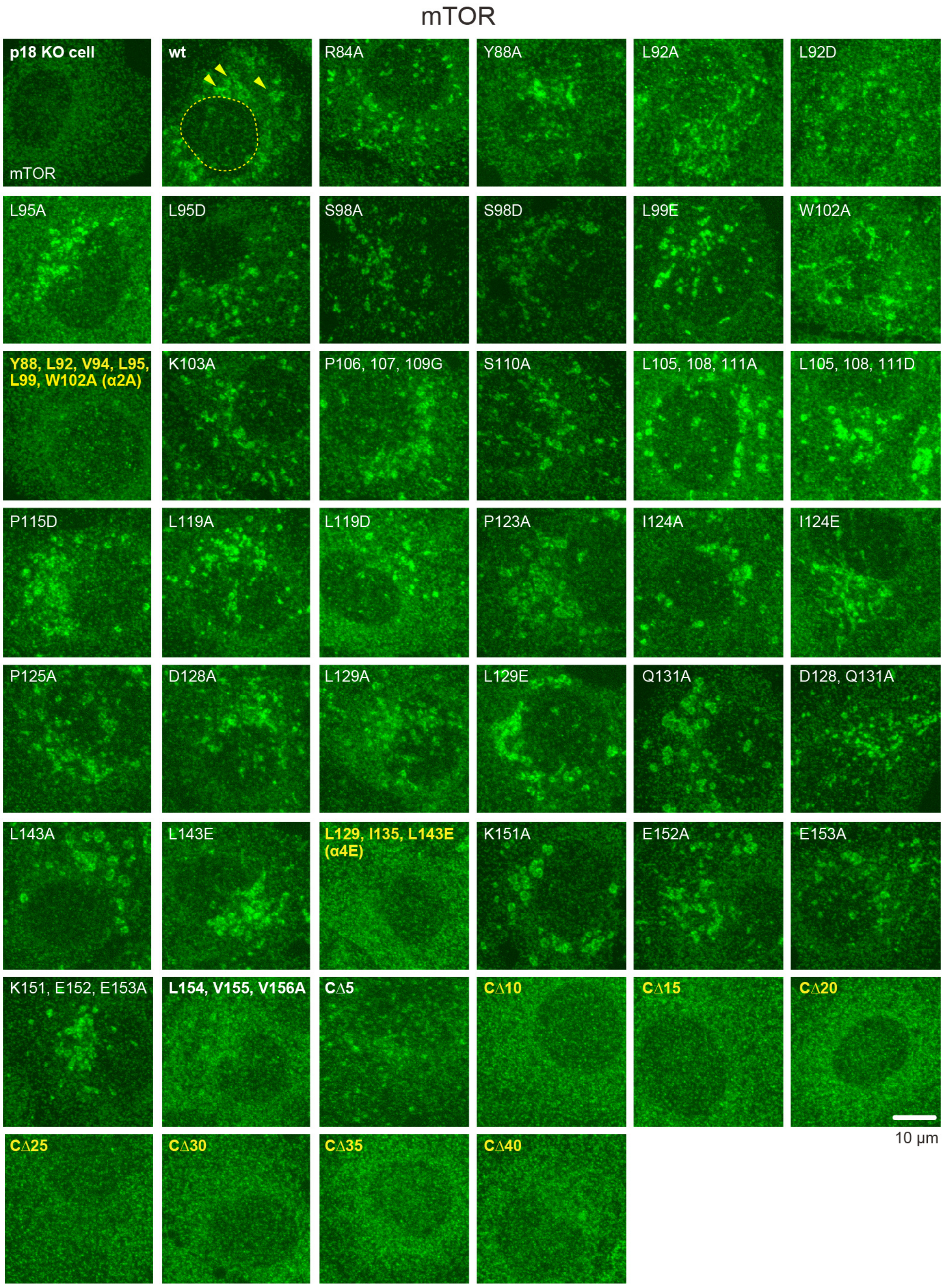

Supplementary Figure 11 | Effects of p18 mutations on the localization of mTORC1.
The intracellular localisations of mTOR in p18 KO cells expressing the indicated p18 mutants was analysed by immunostaining. Arrowheads indicate the punctate distribution of mTOR to perinuclear lysosomes. Dotted lines denote nuclei. p18 mutants that delocalised mTOR are shown in yellow.

Supplementary Figure 12

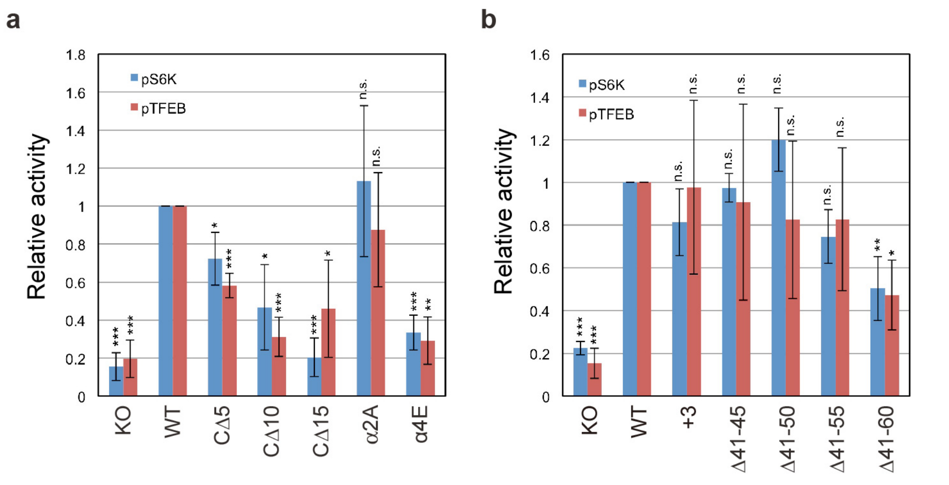

Supplementary Figure 12 | Effects of p18 mutations on the activity of mTORC1.
a, p18 KO cells (KO) were transfected with wild-type p18 (WT), C-terminal deletion mutants (CΔ5, CΔ10, CΔ15), and mutants with multiple substitutions in the α2 helix (α2A) and the α4 helix (α4E), and total cell lysates were subjected to western blotting for pS6K and TFEB. The band intensities of pS6K and phosphorylated form of TFEB were quantified, and values relative to actin are shown. Values represent the mean ± SD (n=3, t-test ***p<0.001, **p <0.01, *p <0.05, n.s.: not significant). **b,** p18 KO cells (KO) were transfected with wild-type p18 (WT) and N-terminal deletion mutants of p18, and total cell lysates were subjected to western blotting for pS6K and TFEB. The band intensities of pS6K and phosphorylated form of TFEB were quantified, and values relative to actin are shown. Values represent the mean ± SD (n=3, t-test ***p<0.001, **p <0.01, *p <0.05, n.s.: not significant).

Supplementary Figure 13

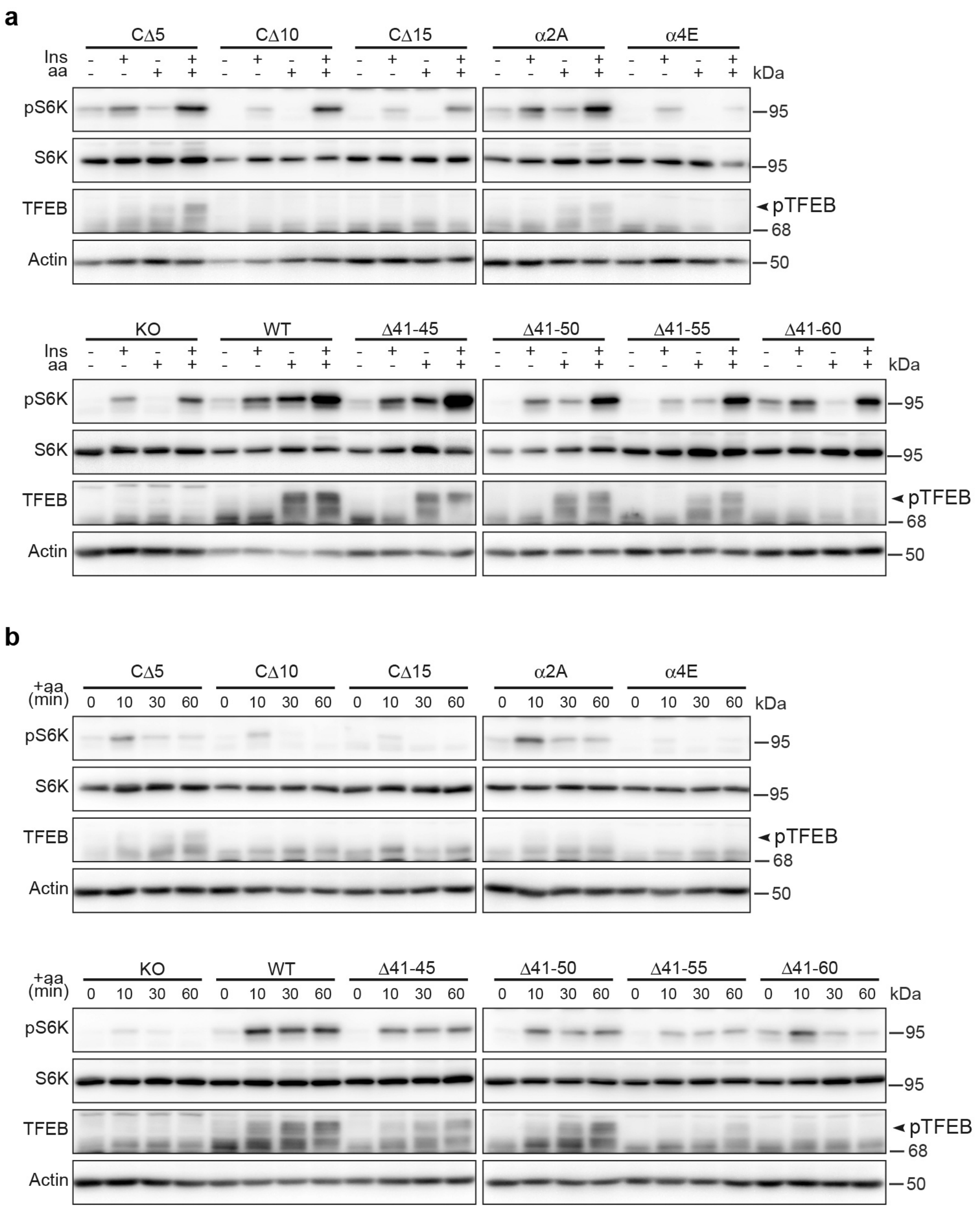

Supplementary Figure 13 | Insulin- and amino acid-dependent activity of mTORC1 in p18 KO cells expressing various p18 mutants.
a, p18 KO cells expressing the indicated p18 mutants were cultured in the medium without amino acids for 1h, then incubated for 1h in the medium containing amino acids and/or insulin. Total cell lysates were subjected to western blotting for the indicated proteins. Arrowheads indicate the locations of phosphorylated (pTFEB) and non-phosphorylated (TFEB) forms of TFEB. **b**, p18 KO cells expressing the indicated p18 mutants were incubated in the medium without amino acids for 1h, then treated with amino acids for the indicated periods. Total cell lysates were subjected to western blotting for the indicated proteins.

Supplementary Figure 14

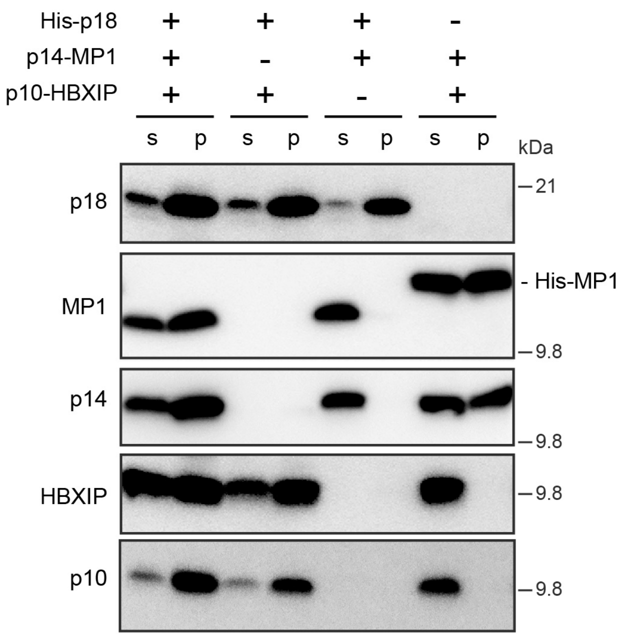

Supplementary Figure 14 | Step-wise formation of Ragulator.
E. coli cells expressing the indicated combinations of His-tagged p18, p14-MP1 (or His tagged MP1) and p10-HBXIP were lysed, and the complex was precipitated with HisTrap beads. Soluble lysates (s) and the precipitates (p) were subjected to western blotting for the indicated proteins.

Supplementary Figure 15

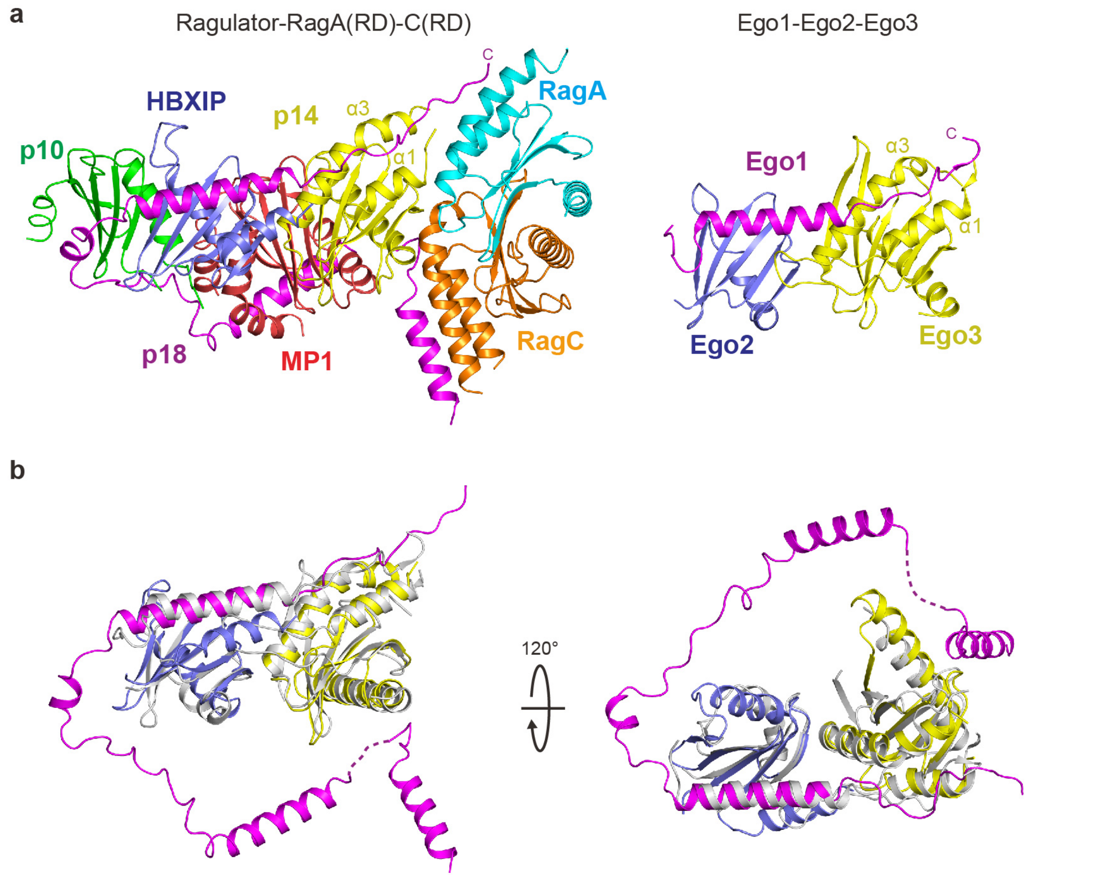

Supplementary Figure 15 | Structural comparison between Ragulator- RagA(RD)-C(RD) and Ego1-Ego2-Ego3 ternary complexes.
a, Ribbon models of the Ragulator-RagA(RD)-C(RD) complex (left panel) and Ego1-Ego2-Ego3 ternary complex (right panel). **b,** Ribbon model of the Ego1-Ego2-Ego3 ternary complex superimposed on the Ragulator-RagA(RD)-C(RD) complex. The superimposed structure of p18-HBXIP-p14 and the Ego1-Ego2-Ego3 ternary complex is viewed from different angles rotated 120° along the horizontal axis (right panel).

Supplementary Figure 16

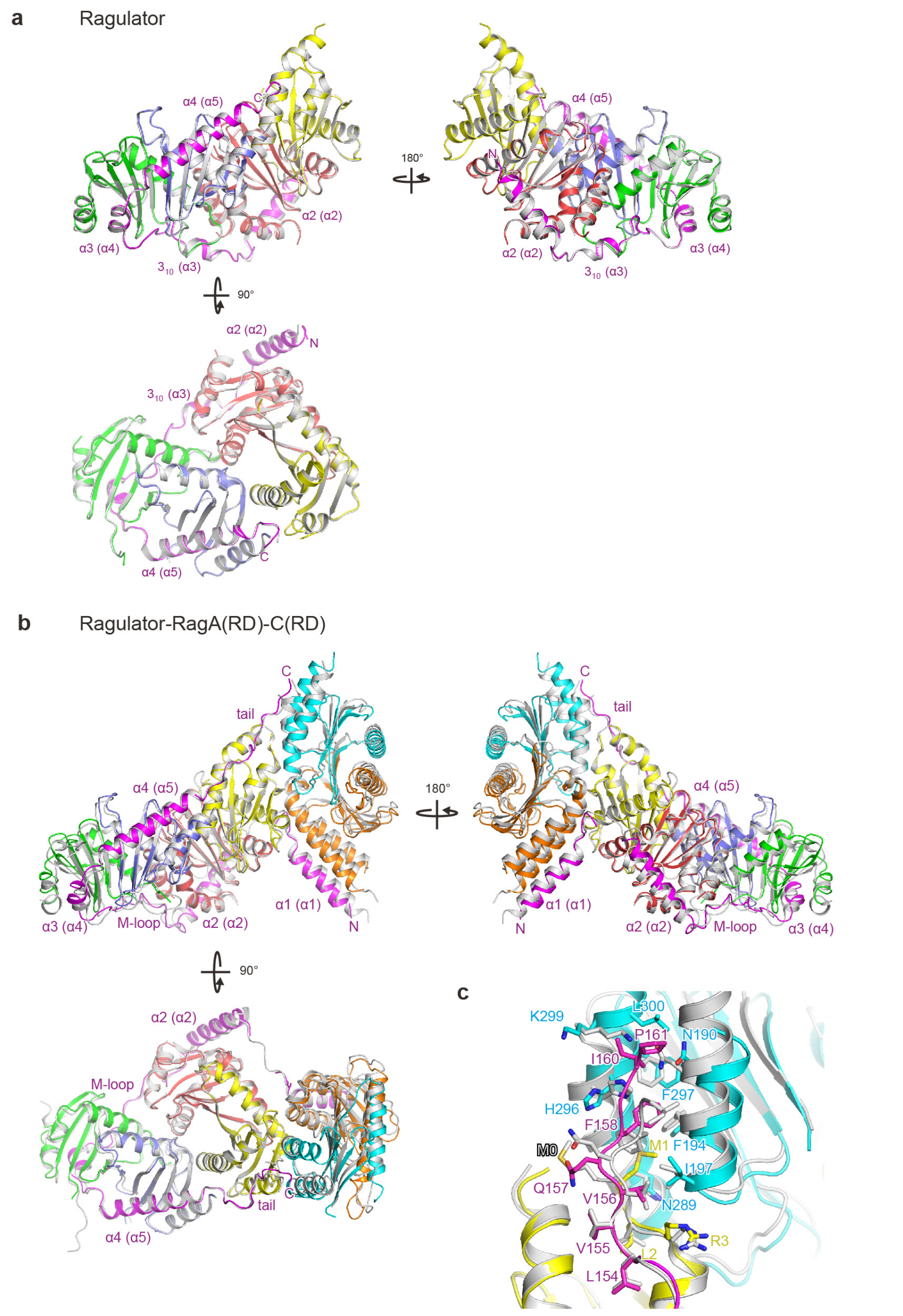

Supplementary Figure 16 | Comparisons between our structures and those by de Araujo et al.
a, Ribbon model of our Ragulator superimposed on that reported by de Araujo et al., which is shown in grey. The structures are viewed from different angles rotated 180° along the vertical axis (right panel) and 90° along the horisontal axis (lower panel). The name of helix in de Araujo's structure is indicated in the parenthesis. **b**, Ribbon model of our Ragulator-RagA(RD)-C(RD) complex superimposed on that reported by de Araujo et al., which is shown in grey. The structures are viewed from different angles rotated 180° along the vertical axis (right panel) and 90° along the horisontal axis (lower panel). **c,** A magnified view of the interaction site between the C-terminal tail of p18 and RagA(RD). The structure by de Araujo et al. is shown in grey.

Supplementary Figure 17

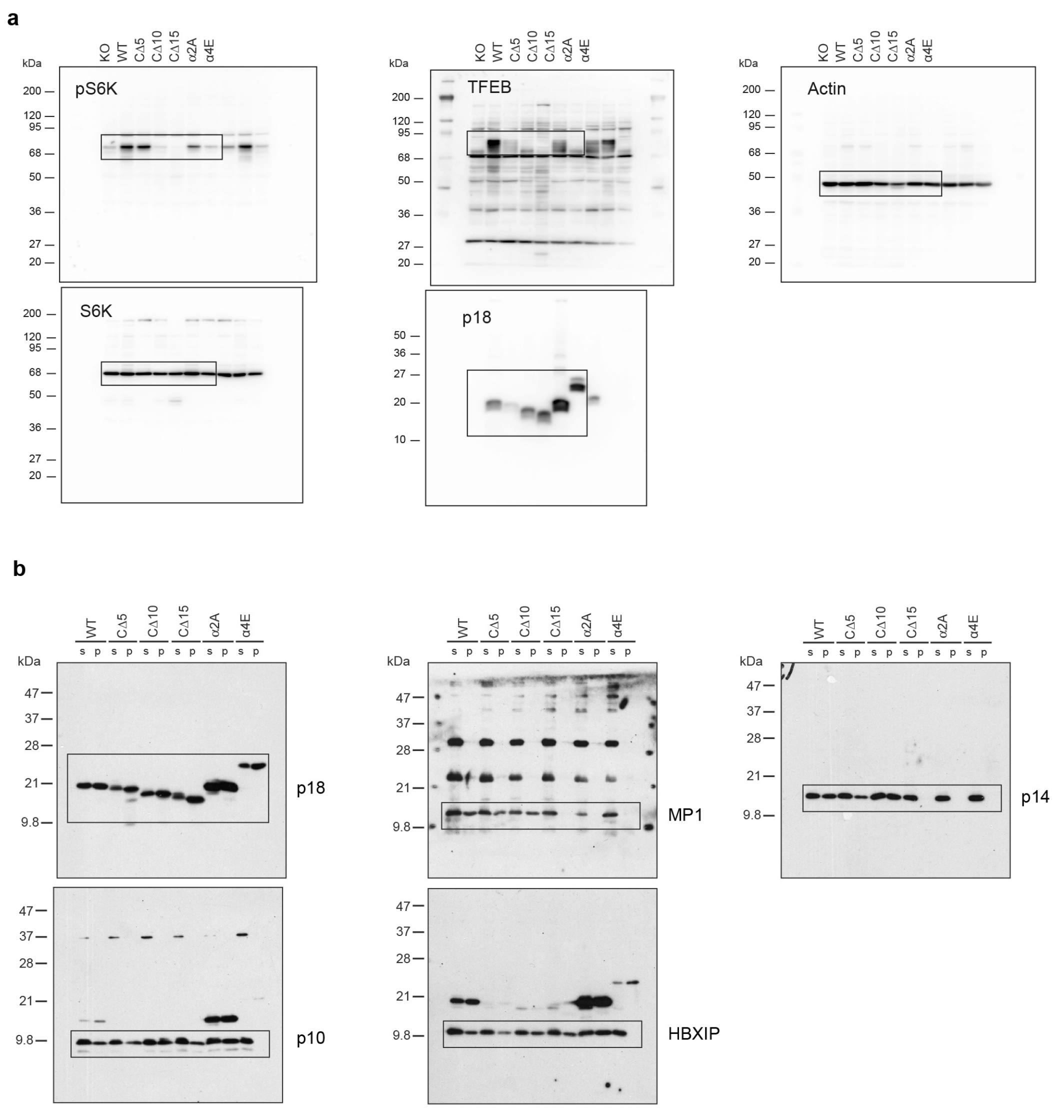

Supplementary Figure 17| Uncropped images of western blots for Figure 4d (a) and 4e (b).

Supplementary Figure 18

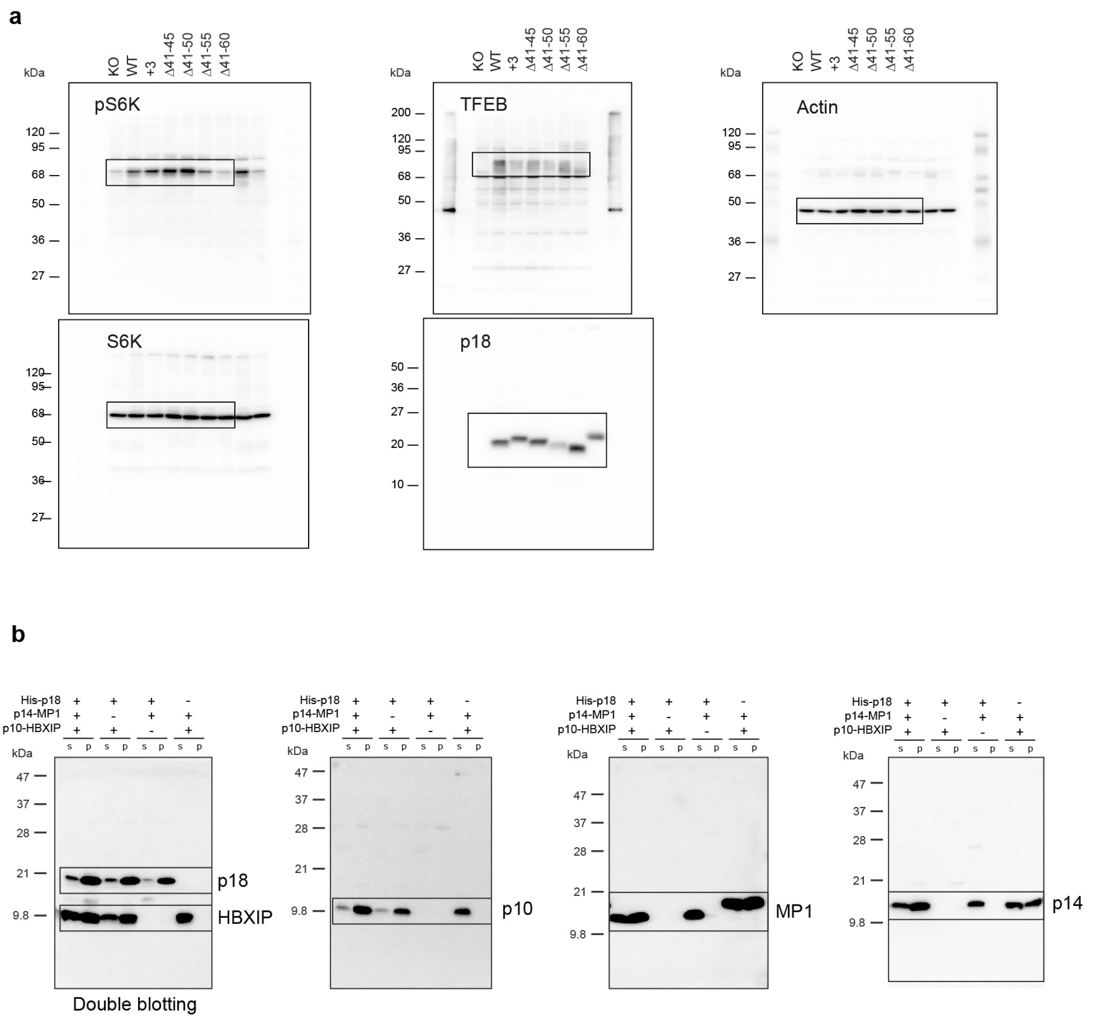

Supplementary Figure 18 | Uncropped images of western blots for Figure 5d (a) and Supplementary Figure 14 (b).

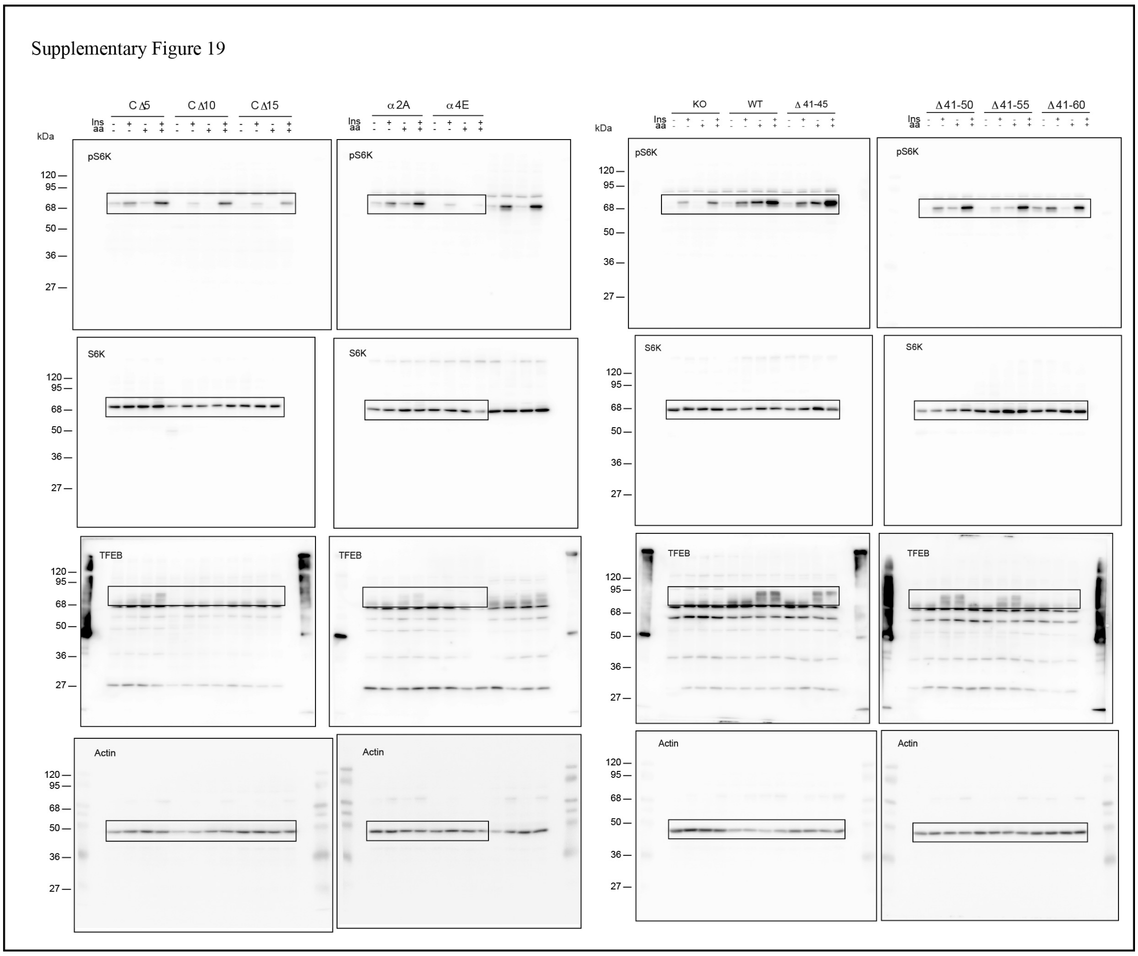

Supplementary Figure 19 | Uncropped images of western blots for Supplementary Figure 13a.

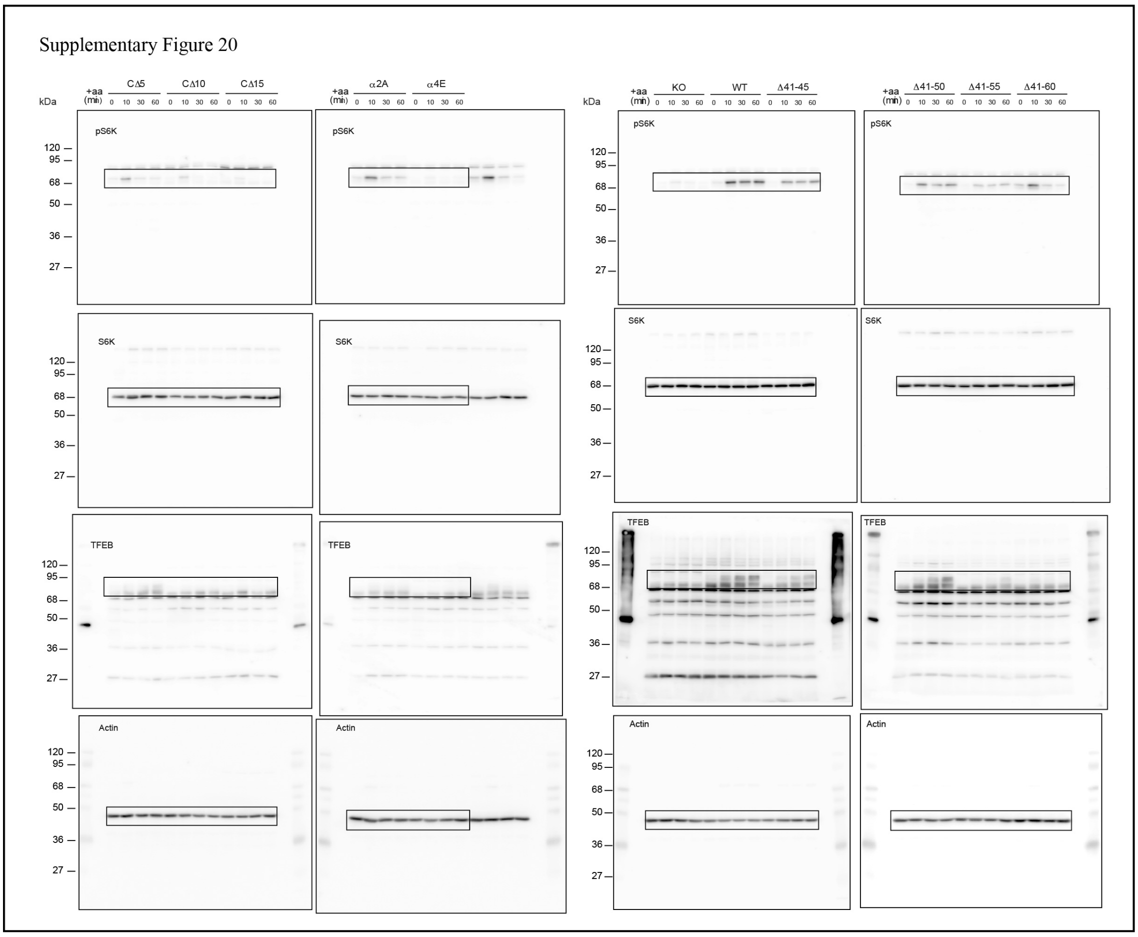

Supplementary Figure 20 | Uncropped images of western blots for Supplementary Figure 13b.

Supplementary Table 1 | Buried surface area (Å^2) between components in Ragulator

	p14	MP1	p10	HBXIP
p18	271.2	758.1	1130.0	922.5
p14		1248.4	No interaction	437.5
MP1			239.5	359.1
p10				829.8

Supplementary Table 2 | Buried surface area (Å^2) between components in the Ragulator-RagA(RD)-C(RD) complex

	p14	MP1	p10	HBXIP	RagA	RagC
p18	769.7	1103.9	954.4	922.8	302.0	771.5
p14		1358.0	No interaction	423.4	643.6	301.2
MP1			32.3	393.5	No interaction	46.3
p10				959.8	No interaction	No interaction
HBXIP					No interaction	No interaction
RagA						1300.4

Supplementary Table 3 | Comparison of crystal structures of Ragulator and Ragulator-RagA(RD)-C(RD) complex

	Ragulator		Ragulator-RagA(RD)-C(RD)	
PDB ID	5X6U	6EHP	5X6V	6EHR
Resolution (Å)	2.40	2.30	2.02	2.90
Visible residues in the crystal structure				
p18/LAMTOR1	Gln78–Ala150	Glu80–Asp149	Asp49–Ala70<br>Met76–Pro161	Ala46–Ile160
p14/LAMTOR2	Pro4–Gly60<br>Glu66–Ser125	Pro4–Gln62<br>Asp67–Val122	Met1–Ser125	Leu2–Ser125
MP1/LAMTOR3	Ala2–Val123	Met1–Glu122	Ala2–Glu122	Asp3–Glu122
p10/LAMTOR4	Thr2–Glu95	Ser3–Arg94	Ala4–His56<br>Val61–Arg92	Met1–Ile97
HBXIP/LAMTOR5	Met1–Ser91	Met1–Ser91	Met1–Ser91	Met1–Ser91
Human RagA	-	-	Leu187–Cys219<br>Arg223–Lys307	Gln182–Cys219<br>Phe228–Lys299
Mouse RagC	-	-	Gln238–Gly369	-
Human RagC	-	-	-	Gly238–Thr372

Supplementary Table 4. List of PCR primers

Primer names	Sequences from 5’-end
p18HisEcoTEV	GCGAATTCGGAAAACCTGTATTTCCAGGGAGCAGCCAGCCTGCCTTCC GCTCGC
p18Not	ATATAGCGGCCGCTCATGGGATCCCAAACTGTAC
S98DF	GCTTGGCTGTGCTGAGCAGCGCCCTGACCCATTGGAAGAA
S98DR	TTCTTCCAATGGGTCAGGGCGCTGCTCAGCACAGCCAAGC
MPNco	TATACCATGGGCAGCAGCGCGGATGACCTAAAGCGATTCTTG
MPHind	CGCAAGCTTAAGAAACTTCCACAACTTGTCT
p14Nde	TATACATATGCTGCGCCCCAAGGCTTTGACC
p14Xho	AGACTCGAGTTAAGATGCCGCCACTTGGGTGAG
HBNco	ATACCATGGAGGCGACCTTGGAGCAGCAC
HBHind	CGCAAGCTTTCAAGAGGCCATTTTGTGCACTGC
p10Nde	ACGCATATGACTTCTGCGCTGACCCAGGGG
p10Xho	AGACTCGAGTCAGACATCAATGGGCTCCCGACC
RagA(RD)Bam	GCGGATCCCAACGTTCAGCAGCTGGAGATG
RagA(RD)Not	TATGCGGCCGCTCAGGGAATCAGCTGGTAGACGAT
RagC(RD)Nde	ATACATATGCAACTGCCCACTTTGGAAAAC
RagC(RD)Xho	CGCTCGAGTCAGCTTCTGTGAGAAGTCACACC
RagABam	CCAGGATCCAAATACAGCCATGAAGAAAAAG
RagANot	TATGCGGCCGCTCAACGCATAAGGAGACTGTGCTT
RagCBgl	GGCAGATCTCTCCCTGCAGTACGGGGCGGAGGAG
RagCXhol	AGACTCGAGCTAGATGGCGTTTCGTGGCGTGCC
R84A	CATGAGTACATGGACGCTGCCAGGCAGTACAGC
Y88F	GACCGTGCCAGGCAGGCTAGCACCCGCTTGGCT
L92A	TACAGCACCCGCGCCGCTGTGCTGAGC
L92D	TACAGCACCCGCGACGCTGTGCTGAGC
V94A	ACCCGCTTGGCTGCTCTGAGCAGCAGC
L95A	CGCTTGGCTGTGGCCAGCAGCAGCCTG
L95D	CGCTTGGCTGTGGACAGCAGCAGCCTG
L99E	CTGAGCAGCAGCGAGACCCATTGGAAG
W102A	AGCCTGACCCATGCCAAGAAGCTGCCA
K103A	AGCCTGACCCATTGGGCCAAGCTGCCACCGCTG
L105A	ACCCATTGGAAGAAGGCCCCACCGCTGCCGTCT
L105D	ACCCATTGGAAGAAGGACCCACCGCTGCCGTCT
P106/107/109G	TGGAAGAAGCTGGGAGGGCTGGGGTCTCTTACCAGC
S110A	CCACCGCTGCCGGCCCTTACCAGCCAG
L105/108/111A	CATTGGAAGAAGGCCCCACCGGCCCCGTCTGCCACCAGCCAGCCC
L105/108/111D	CATTGGAAGAAGGACCCACCGGACCCGTCTGACACCAGCCAGCCC
P115G	CTTACCAGCCAGGGCCACCAAGTGCTG
L119A	CCCCACCAAGTGGCCGCCAGTGAGCCC
L119D	CCCCACCAAGTGGACGCCAGTGAGCCC
P123A	CTGCCGTCTCTTGCCAGCCAGCCCCAC
I124A	CTGGCCAGTGAGCCCGCCCCGTTCTCTGATTTG
I124E	CTGGCCAGTGAGCCCGAGCCGTTCTCTGATTTG
P125A	GCCAGTGAGCCCATCGCCTTCTCTGATTTGCAG
D128A	ATCCCGTTCTCTGCTTTGCAGCAGGTCTCCAGGATAGCT
L129A	ATCCCGTTCTCTGATGCCCAGCAGGTCTCCAGG
L129E	ATCCCGTTCTCTGATGAGCAGCAGGTCTCCAGG
Q131A	ATCCCGTTCTCTGATTTGCAGGCTGTCTCCAGGATAGCT
D128A/Q131A	ATCCCGTTCTCTGCTTTGCAGGCTGTCTCCAGGATAGCT
I135E	CAGCAGGTCTCCAGGGAGGCTGCTTATGCCTAC
L143A	ACGGATCTGAGAGCCTGCACTGTAGGC
L143E	ACGGATCTGAGACTCTGCACTGTAGGC
K151A	ATCCGTGTGGACGCAGCCGAGGAGCTGGTTGTA
E152A	CGTGTGGACGCAAAAGCCGAGCTGGTTGTACAG
E153A	GTGGACGCAAAAGAGGCCCTGGTTGTACAGTTT
K151A/E152A/E153 A	TATGCGGCCGCTCATGGGATCCCAAACTGTACAACCAGGGCAGCAGC TGCGTCCAC
L154A	CGCAAAAGAGGAGGCCGTTGTACAGTTTG
V155A	GTGCACGAACGCGTTGGGATCCCAAACTGTACGGCCAGCTCCTCTTTT

	GCGTC
V156A	GTGCACGAACGCGTTGGGATCCCAAACTGGGCAACCAGCTCCTCTTTT GC
p18D5	TAGGCGGCCGCTCATACAACCAGCTCCTCTTTTGCGTC
p18D10	TAGGCGGCCGCTCATTTTGCGTCCACACGGATCTGAGA
p18D15	TAGGCGGCCGCTCAGATCTGAGAAAGTGCACTGTAGGC
p18D20	TAGGCGGCCGCTCAACTGTAGGCATAAGCAGCTATCCT
p18D25	TAGGCGGCCGCTCAAGCTATCCTGGAGACCTGCTGCAA
p18D30	TAGGCGGCCGCTCACTGCTGCAAATCAGAGAACGGGAT
p18D35	TAGGCGGCCGCTCAGAACGGGATGGGCTCACTGGCCAG
p18D40	TAGGCGGCCGCTCAACTGGCCAGCACTTGGTGGGGCTG
p18HisTevD45	GCGAATTCGGAAAACCTGTATTTCCAGGGAGCAGCCCGCACTGATGA GCAGGCC
p18HisTevD50	GCGAATTCGGAAAACCTGTATTTCCAGGGAGCAGCCGCCCTGCTCTCT TCCATC
p18HisTevD55	GCGAATTCGGAAAACCTGTATTTCCAGGGAGCAGCCATCCTTGCCAA GACAGCC
p18HisTevD60	GCGAATTCGGAAAACCTGTATTTCCAGGGAGCAGCCGCCAGCAACAT CATTGAT
Eco-p18-f	TGAATTCACCATGGGGTGCTGCTACAGCAG
p18N40-Bam-r	TGGATCCGCGTAGTTGGGCTCGGCTCCATT
p18-NotI-r	CTCGCGGCCGCTCATGGAATCCCAAACTGTA
p18BamHI-N41	GGGATCCTCACAGCCTGCCTTCCGCTCG
p18BamHI-N46	GGGATCCTGCTCGCACTGATGAGCAGGC
p18BamHI-N51	GGGATCCTCAGGCCCTGCTCTCTTCCAT
p18BamHI-N56	GGGATCCTTCCATCCTTGCCAAGACAGC
p18BamHI-N61	GGGATCCTACAGCCAGCAACATCATTGA

Supplementary Table 5 | Dilutions of antibodies.

Antigen	Supplier	Catalog#	Application	Dilution (-fold)
p18(LAMTOR1)	Cell signaling Technology	8975S	WB	1,000
			IF	1,000
p14(LAMTOR2)	Cell signaling Technology	8145	WB	2,000
MP1(LAMTOR3)	Cell signaling Technology	8168	WB	2,000
p10(LAMTOR4)	Cell signaling Technology	12284	WB	2,000
HBXIP(LAMTOR5)	Cell signaling Technology	14633	WB	2,000
mTOR	Cell signaling Technology	2983S	IF	1,000
p-S6K	Cell signaling Technology	9234	WB	1,000
TFEB	Bethyl Laboratories Inc	A303-673A	WB	2,000
Actin	Santa Cruz Biotechnology	sc-1615	WB	1,000
LAMP1	Santa Cruz Biotechnology	sc-19992	IF	1,000
S6K	Santa Cruz Biotechnology	sc-8418	WB	2,000

WB; western blotting, IF; immunofluorescence
