# Supplementary material for: Structural basis for the assembly of the Ragulator-Rag GTPase complex
Source: Nat Commun. 2017 Nov 20;8:1625. doi: 10.1038/s41467-017-01762-3 (PMC5696360; doi:10.1038/s41467-017-01762-3)
Supplement: Supplementary file 2 — Description of Additional Supplementary Files [file 41467_2017_1762_MOESM2_ESM.pdf]

Description of Supplementary Files

File Name: Supplementary Movie 1
Description: **3D visualisation of the structure of the Ragulator - RagA(RD)-C(RD) complex.** p18, magenta; p14, yellow; MP1, red; p10, green; HBXIP, blue; RagA(RD), cyan; RagC(RD), orange.
